# Atomic-level structural responsiveness to environmental conditions from 3D electron diffraction

**DOI:** 10.1038/s41467-022-34237-1

**Published:** 2022-11-04

**Authors:** Yang Ling, Tu Sun, Linshuo Guo, Xiaomeng Si, Yilan Jiang, Qing Zhang, Zhaoxi Chen, Osamu Terasaki, Yanhang Ma

**Affiliations:** 1 School of Physical Science and Technology, ShanghaiTech University, Shanghai, 201210 PR China; 2 Shanghai Key Laboratory of High-resolution Electron Microscopy, ShanghaiTech University, Shanghai, 201210 PR China; 3 Center for Transformative Science, ShanghaiTech University, Shanghai, 201210 PR China

**Keywords:** Characterization and analytical techniques, Metal-organic frameworks

## Abstract

Electron microscopy has been widely used in the structural analysis of proteins, pharmaceutical products, and various functional materials in the past decades. However, one fact is often overlooked that the crystal structure might be sensitive to external environments and response manners, which will bring uncertainty to the structure determination and structure-property correlation. Here, we report the atomic-level ab initio structure determinations of microcrystals by combining 3D electron diffraction (3D ED) and environmental transmission electron microscope (TEM). Environmental conditions, including cryo, heating, gas and liquid, have been successfully achieved using in situ holders to reveal the simuli-responsive structures of crystals. Remarkable structural changes have been directly resolved by 3D ED in one flexible metal-organic framework, MIL-53, owing to the response of framework to pressures, temperatures, guest molecules, etc.

## Abstract (web summary)

Materials’ structure of are often sensitive to external stimuli, which will bring uncertainty to the structure determination. Here authors present a 3D electron diffraction approach to study the stimuli responsive structures of crystals with atomic precision under various environmental conditions.

## Introduction

Properties of matters are intrinsically dependent on the internal arrangement of atoms or molecules, and therefore, the knowledge of their three-dimensional crystal structure is a prerequisite for structure-property correlations and further design of functional materials^1–3^. In particular, many stimuli-responsive materials feature a phase transition or structural changes that can be triggered by external environmental conditions^4,5^. Three-dimensional structure solution under various environmental conditions is essential to identify the native state of materials and provide insights into their functions. However, the atomic-level structural analysis of single crystals responsive to external stimuli is rarely achieved, which brings ambiguity to the phase identification and structure-property relationship studies. Therefore, new methodological developments are expected to discover the atomic-level structural responses to various environments.

3D ED is an emerging technique for single crystal structure analysis from sub-micro/nanosized crystals using high-energy electrons in the past decade, which has been widely used for structural analysis of inorganic, organic and protein microcrystals recently^6–13^. However, the studies of structural responsiveness to external stimuli are rarely achieved.

**Fig. 1:**
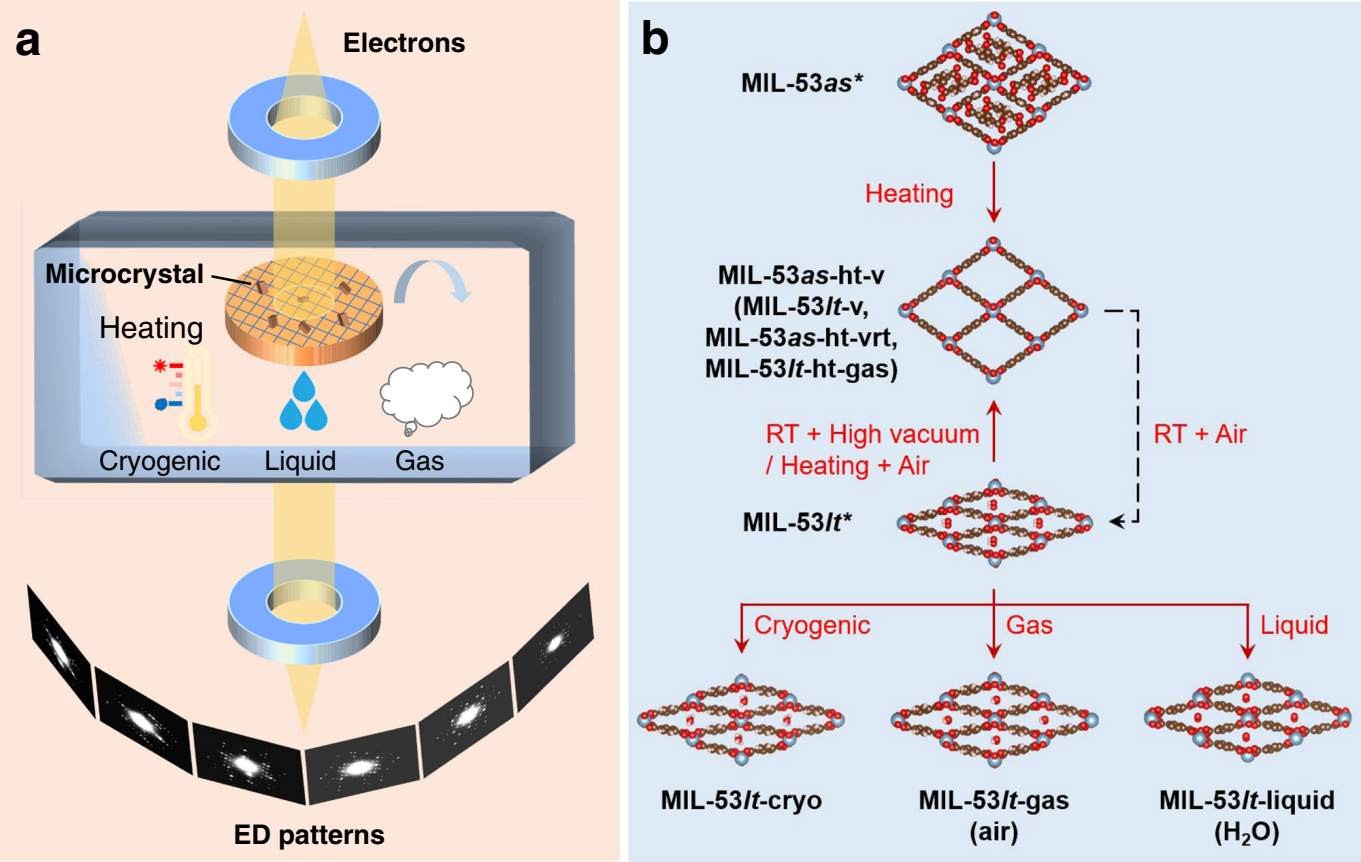
Representation of environmental 3D electron diffraction and its application. **a** The crystals are placed into various environmental conditions, such as heating, cryogenic cooling (after plunge-freezing sample preparation), gas atmosphere and liquid environments, and 3D electron diffraction data are collected. **b** The application of environmental 3D electron diffraction in studying the flexible MOF microcrystals, MIL-53. The symbol * in **b** means the two initial samples prepared for TEM observations. The dashed line indicates a step carried out outside the TEM. Source data are provided as a Source Data file.

In this work, we propose a method, called environmental 3D ED, by placing single microcrystals into various conditions including cryo, heating, gas environments and liquids, and collecting 3D ED data under low electron dose conditions and continuous crystal tilting mode. This combination of techniques overcomes the limits of current 3D ED methods and has been proven to be a powerful method for investigating the structural transitions induced by various stimuli (Fig. 1). Except for normal high vacuum and low-temperature conditions (Supplementary Fig. 1) in transmission electron microscopy, other environmental conditions including heating, gaseous and liquid have been achieved in microchips (Supplementary Figs. 2, 3). A typical flexible metal-organic framework (MOF) material, MIL-53, has been taken as an example to show the application of this method to understand the dynamic behaviors of structures under different environmental conditions.

## Results

MIL-53 is one of the most representative flexible MOFs synthesized with terephthalic acid (or its derivatives) and metal ions (Cr, Al, Fe, Sc and Ga). The MIL-53 sample was prepared by hydrothermal reaction using terephthalic acid and aluminum nitrate in water according to the reported literature^14^. Three phases have been reported previously, the as-made phase (MIL-53*as*), the high-temperature phase (MIL-53*ht*) and the H_2_O-containing phase (MIL-53*lt*, room temperature, in the air) through powder X-ray diffraction (PXRD) analysis. Meanwhile, MIL-53 can form various stable structures after adsorbing different guest molecules (such as CH_4_, CO_2_, etc.)^15–18^.

Scanning electron microscope images show that the crystals have a polyhedral morphology with size in the range of 1–6 micrometers (Supplementary Figs. 4, 5). PXRD results confirm the high crystallinity and the pure phase of MIL-53 (Supplementary Figs. 6, 7). In situ PXRD data were collected from the MIL-53 powder under different temperatures (Supplementary Fig. 8), which clearly indicated the reversible phase transitions or structural changes.

**Fig. 2:**
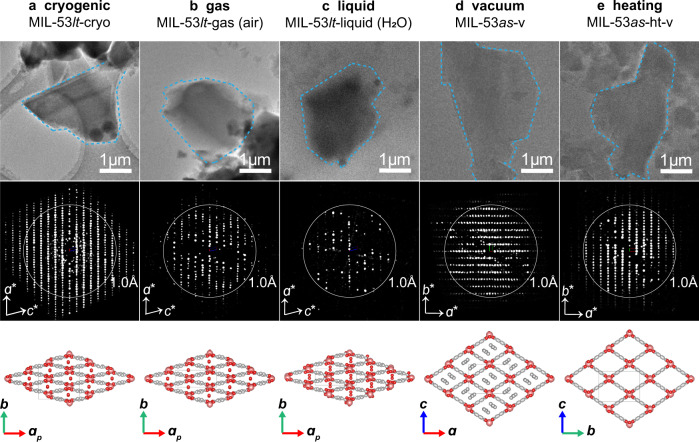
TEM images, environmental 3D ED data and determined structures from single crystals (MIL-53) under different conditions. The 3D ED data are projected along the (**a-c**) [010] and (**d**, **e**) [001] directions. Each projection is from a single 3D ED dataset without data merging. **a** MIL-53*lt*-cryo: the low temperature phase prepared by plunge-freezing and cryogenic transfer protocols. **b** MIL-53*lt*-gas (air): MIL-53*lt* in the static air (0.1 MPa). **c** MIL-53*lt*-liquid (H_2_O): MIL-53*lt* covered with liquid water. **d** MIL-53*as*-v: the as-synthesized phase MIL-53*as* under high vacuum state. **e** MIL-53*as*-ht-v: the phase after calcination of MIL−53*as* at 603 K and high vacuum for 2 h. Particles marked with blue dashed lines were used for collecting 3D ED data. White circles in diffraction data represent the resolution of 1 Å. Source data are provided as a Source Data file.

**Fig. 3:**
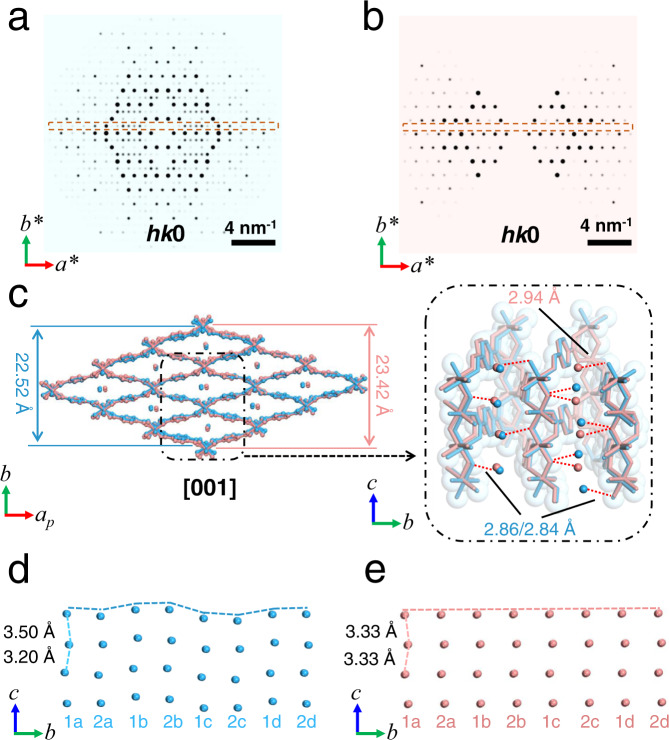
Comparison of two structures obtained from crystals under different environments. The slices at *hk*0 plane cut from reconstructed reciprocal lattice of data of (**a**) MIL-53*lt*-cryo and (**b**) MIL-53*lt*-gas (air). Compared with **b**, extra reflections can be clearly observed in **a** as shown in the dashed boxes, indicating the structural change after the cryogenic sample preparation. For a better display, reflection intensities are 0.8 power of original values. **c** Overlap of two structures of MIL-53*lt*-cryo (blue) and MIL-53*lt*-gas (air) (pink). The inset shows possible hydrogen bonds between oxygen sites (guest H_2_O molecules) and bridging oxygen atoms in the framework, distances of which are 2.86 Å or 2.84 Å for MIL-53*lt*-cryo and 2.94 Å for MIL-53*lt*-gas (air). The frameworks and sites of H_2_O in the channels are slightly different. *a*_*p*_ indicates the projected direction of the *a*-axis. **d**, **e** The different arrangements of guest H_2_O molecules in two structures are displayed further with layers perpendicular to [100] direction. The distances between the nearest guest H_2_O molecules are 3.20 Å or 3.50 Å for MIL-53*lt*-cryo and 3.33 Å for MIL-53*lt*-gas (air) along [001] direction. Two nearest columns of H_2_O sites in the same layer (like 1a – 1b, 2a – 2b) stagger with each other for MIL-53*lt*-cryo, which is different from MIL-53*lt*-gas (air). Source data are provided as a Source Data file.

After removal of the free terephthalic acid molecules inside pores by calcination, MIL-53 can very readily adsorb H_2_O molecules from the air and form the MIL-53*lt* phase. When the sample was directly placed in the high-vacuum TEM column (<1 × 10^−5^ Pa) at room temperature, the resulted phase (MIL-53*lt*-v, *a* = 6.69(3) Å, *b* = 17.11(4) Å, *c* = 12.21(4) Å, *α* = *β* = *γ* = 90°, space group *Imma*, Supplementary Fig. 9a–c) is similar to the reported MIL-53*ht*^14^ with empty channels, rather than MIL-53*lt* with guest H_2_O molecules. Moreover, slight diffuse streaks along the c^*^ axis (Supplementary Fig. 9b) indicate the possible existence of defects in the structure. To stabilize the guest molecules H_2_O inside the pores, we first used plunge-freezing and cryogenic transfer protocols in the TEM sample preparation (MIL-53*lt*-cryo, Fig. 2a). From the collected 3D ED data under these conditions, the lattice parameters were determined to be *a* = 19.55(8) Å, *b* = 15.01(3) Å, *c* = 6.655(10) Å, *α* = *γ* = 90°, *β* = 103.7(2) °, *V* = 1898(9) Å^3^, and space group *P*2_1_/n (Table 1 and Supplemental Figs. 10, 11). It turns out that the observed phase is different from the reported MIL-53*lt* phase^14^ too. Extra reflections appear between two rows of strong reflections, as shown in 2D slice of *hk*0 plane from reconstructed reciprocal lattice (Fig. 3a and Supplementary Fig. 10), which means the double of unit cell parameter *b* compared to the reported one (7.612 Å) and the change of symmetry. Structure solution and refinement were carried out from the 3D ED data (Supplementary Fig. 10a–c and Supplementary Tables 1, 2). Each unit cell contains eight sites of H_2_O molecule in the channels. The distance between two adjacent sites of oxygen atoms (H_2_O) inside the channels is 3.198(7) Å or 3.498(7) Å, respectively. And the separation from each oxygen atom sites to its two closest oxygen atoms in the framework is 2.842(8) Å or 2.857(8) Å, respectively (Fig. 3c), which indicates a possible existence of hydrogen bonds. Two nearest columns of H_2_O molecules in the same layer perpendicular to [100] direction stagger with each other (like 1a-1b, or 2a-2b) along [001] direction (Fig. 3d). At the same time, the orientations of [AlO_6_] octahedra also slightly change (Supplementary Fig. 12a). Both contribute to the double of cell parameter *b*. These results demonstrate that the configuration of the framework and channels is closely related to the host-guest interactions between the framework and H_2_O molecules. The possible effects from ultrasound sonication during the sample preparation were excluded by a control experiment (Supplementary Fig. 13).

**Table 1:** Sample preparation procedures, space group and unit cell parameters of different phases of MIL-53 obtained from environmental 3DED data

	Sample preparation procedure	Space group	Cell parameters (*a*/Å, *b*/Å, *c*/Å, *β*/°)
MIL-53*lt*-v	MIL-53*lt* in vacuum	*Imma*	6.69(3), 17.11(4), 12.21(4), 90.0
MIL-53*lt*-cryo	MIL-53*lt* prepared via the plunge-freezing protocol	*P*2_1_*/n*	19.55(8), 15.01(3), 6.655(10), 103.7(2)
MIL-53*lt*-v-cryo	MIL-53*lt* in vacuum and then cooled by liquid nitrogen	*/*	6.62, 20.54, /, /
MIL-53*lt*-gas (air)	MIL-53*lt* in ambient air and 298 K	*Cc*	19.55(2), 7.81(2), 6.62(2), 104.5(2)
MIL-53*lt*-liquid (H_2_O) *	MIL-53*lt* covered with liquid water	*Cc*	19.54, 7.62, 6.56, 105.0
MIL-53*as*-v	MIL-53*as* in vacuum	*Pnma*	17.03(1), 6.57(2), 12.16(2), 90.0
MIL-53*as*-ht-v	MIL-53*as* calcined at 603 K in vacuum for 2 h	*Imma*	6.61(3), 17.25(3), 12.81(1), 90.0
MIL-53*as*-ht-vrt	MIL-53*as*-ht-v back to 298 K	*Imma*	6.65(3), 17.27(3), 12.54(5), 90.0
MIL-53*lt*-ht-gas	MIL−53*lt* is heated at 603 K in ambient air for 2 h	*Imma*	6.62(2), 16.73(4), 12.89(4), 90.0

Note: * means the complete reflection conditions cannot be summarized from experimental 3DED data due to the low completeness. The space group is assumed to be same as MIL-53*lt*-gas (air).

We also tried to carry out the sample cooling process using liquid nitrogen after inserting the holder into the TEM column, without the plunge-freezing sample preparation and cryo-transfer in this case. 3D ED results (Supplementary Fig. 9d–f) show that the obtained structure MIL-53*lt*-v-cryo is different from MIL-53*lt*-v and MIL-53*lt*-cryo. The unit cell parameter *b* of this phase (Table 1, *b* = 20.54 Å) is longer than that of room temperature phase MIL-53*lt*-v (*b* = 17.11 Å), which may be caused by the temperature-induced transition from large pores to low-temperature narrow pores^19,20^. Moreover, diffuse streaks become more serious along the c* axis than that in MIL-53*lt*-v (Supplementary Fig. 9), which represent severer disorders in the structure after the slow cooling process.

To be close to the actual working conditions of materials, gas environment was introduced into the 3D ED data collection of MOFs. Static air (0.1 MPa) was sealed in an in situ gas cell to create the gas environment around the MIL-53 crystals (MIL-53*lt*-gas (air), Fig. 2b). Leak check was performed before being inserted into the TEM. Notably, the diffraction spots disappeared too fast to collect a 3D ED dataset under high-energy electron beam irradiation (~0.2 e^−^Å^−2^s^−1^), showing that MIL-53 crystals suffered severe beam damage from the ionized gas by high-energy electrons. Notably, the damage was remarkably reduced under lower dose conditions (~0.045 e^−^Å^−2^s^−1^). Meanwhile, every single set of 3D ED data was collected efficiently within one minute. The completeness of a single dataset is usually below 40%, due to the limited tilting angles of in situ holders inside the TEM. Therefore, merging of multiple datasets to improve the completeness is necessary (Supplementary Fig. 14). 3D ED data were collected from micrometer sized crystals and ten high-quality datasets were merged, giving a resolution of 0.80 Å and completeness of 70.8%. The lattice parameters of MIL-53*lt*-gas (air) were determined to be *a* = 19.55(2) Å, *b* = 7.81(2) Å, *c* = 6.62(2) Å, *α* = *γ* = 90°, *β* = 104.5(2) °, *V* = 978(4) Å^3^ (Table 1) with space group of *Cc*, which is consistent with the reported structure of MIL-53*lt*^14^. In contrast to MIL-53*lt*-cryo, we did not observe extra rows of diffraction spots in the case of MIL-53*lt*-gas (air) (Fig. 3b and Supplementary Fig. 15). The refinement results show the successful solution of the framework and location of the H_2_O guest molecules. A single unit cell contains four sites of H_2_O molecules as guests within the straight channels. The distance between two adjacent sites of oxygen atoms (H_2_O) inside the channels is 3.33(1) Å, and the separation from each oxygen atom site to its two closest oxygen atoms in the framework is 2.94(6) Å, which also indicates a possible existence of hydrogen bonds. Moreover, compared with MIL-53*lt*-cryo, H_2_O molecules in MIL-53*lt*-gas (air) are regularly distributed in all the channels, and the arrangement of [AlO_6_] octahedra is concordant along the [010] direction (Fig. 3e, Supplementary Fig. 12b, and Supplementary Tables 3, 4).

**Fig. 4:**
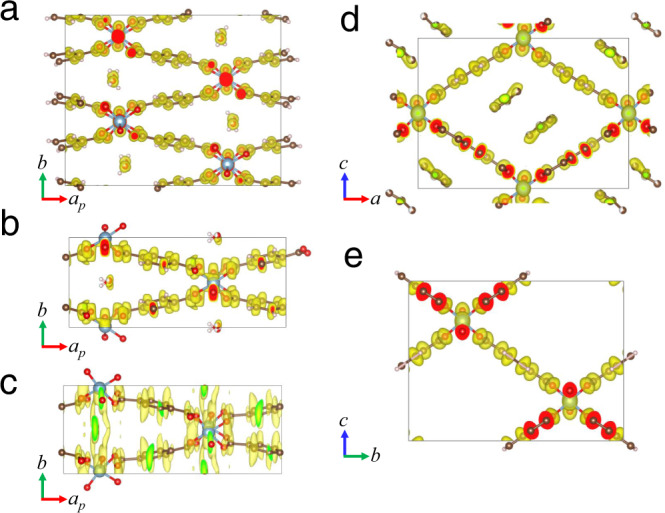
Electrostatic potential maps overlaid with crystal structure models obtained from environmental 3D ED data. **a** MIL-53*lt*-cryo, **b** MIL-53*lt*-gas (air), **c** MIL-53*lt*-liquid (H_2_O), **d** MIL-53*as*-v and **e** MIL-53*as*-ht-v. The structure of MIL-53*lt*-liquid (H_2_O) was solved by the direct-space strategy. Yellow color represents observed potential map while red or green color represent sections between potential map and unit cell boundary. The isosurface level is 2σ[*V*(r)], which is defined in VESTA software as: *d*(iso) = 〈|*V*(r)|〉 + *n* × *σ*(|*V*(r)|), where 〈|*V*(r)|〉 is the average of |*V*(r)|’s, σ(|*V*(r)|) is the standard deviation of |*V*(r)|, and *n* is a parameter to adjust *d*(iso). Source data are provided as a Source Data file.

In addition to gaseous atmosphere, another common environmental condition is liquid solution. We further explored the possibility of 3D ED data collection from microcrystals covered with liquid water, which was achieved using in situ liquid chips and holder (Supplementary Fig. 2). The 3D ED data collection in the liquid environment is much more challenging than that in other environmental conditions, as the liquid give a much stronger scattering and the suspending crystals in the liquid will randomly rotate or drift under beam irradiation (Supplementary Figs. 16, 17 and Supplementary Movies 1, 2). The ionization of the liquid might also cause damages to the crystals. Besides, these micrometer sized crystals need an in situ liquid cell with a large spacer, which simultaneously bring a thick liquid layer. Great efforts had been made to collect 3D ED data under liquid environment. Finally, we succeeded in collecting one dataset which was used to determine the unit cell *a* = 19.54 Å, *b* = 7.62 Å, *c* = 6.56 Å, *α* = *γ* = 90°, *β* = 105° (Table 1 and Supplementary Fig. 18). This phase was identified as MIL-53*lt*-liquid (H_2_O) (Fig. 2c). Structure solution (Fig. 4c and Supplementary Tables 5, 6) was obtained using the direct-space strategy^21^ instead of direct methods, but further structure refinement was not carried out due to the low completeness (~21% with the resolution of 1 Å) and low signal-to-noise ratio of the experimental data (Supplementary Table 5). The framework structure of MIL-53*lt*-liquid (H_2_O) is very close to that of MIL-53*lt*-gas (air), which accords with PXRD results (Supplementary Fig. 7). The slight differences of two electrostatic potential maps (Fig. 4) come from the different qualities of 3D ED data. Of note, the small size of particles (less than 300 nm) and a controllable thickness of liquid layer will be beneficial to liquid 3D ED experiments. It is also worth mentioning that the feasibility of this protocol was further proven using LTA https://asia.iza-structure.org/IZA-SC/framework.php?STC=LTA zeolite nanocrystals (Supplementary Figs. 19, 20), which have been widely used in chemical industry for dehydration and separation. All of the Si/Al and O atoms could be successfully located in the ab initio structure solution. The determination of their atomic crystal structures under working conditions will be vital for their structure-property correlation and design of new materials.

When H_2_O molecules exist in the pore of activated MIL-53, the structure is sensitive to high-vacuum and temperature. However, the case may be different for the as-synthesized phase (MIL-53*as*) that contains free terephthalic acid molecules inside the channels. To show the different magnitudes of interaction between the guest molecules and the framework, the grid containing MIL-53*as* crystals was loaded on a high-tilting holder, and inserted into the high-vacuum TEM column (MIL-53*as*-v, Fig. 2d). 3D ED data were successfully collected at room temperature (298 K) and high vacuum (<1 × 10^−5^ Pa). The resolution of a single 3D ED dataset can achieve 0.85 Å with completeness of 60%-85%. Moreover, the completeness was further improved to 98.7% by merging two datasets (see details in the methods). The unit cell parameters were determined to be *a* = 17.03(1) Å, *b* = 6.57(2) Å, *c* = 12.16(2) Å, *α* = *β* = *γ* = 90°, *V* = 1361(5) Å^3^ (Table 1). The well-defined framework can be clearly observed in the ab initio structure solution with space group *Pnma* using direct methods (Fig. 4d, Supplementary Fig. 21 and Supplementary Tables 7–9). The unit cell of MIL-53*as*-v is same as MIL-53*as*, but different from the guest-free MIL-53*ht*^14^, which indicates that guests might still exist in the channels. It is difficult to locate guest terephthalic molecules through structural refinement, probably because these molecules are highly disordered in the channels of MIL-53*as*-v. The amount of free terephthalic acid molecules was confirmed by thermogravimetry analysis (Supplementary Fig. 22). Moreover, we also collected 3D ED data from MIL-53*as* crystals treated with cooling in the vacuum and cryogenic protocol (Supplementary Fig. 23). The determined structures, denoted as MIL-53*as*-v-cryo and MIL-53*as*-cryo respectively, are very close to MIL-53*as*-v.

The removal of free terephthalic acid molecules from the channels of MIL-53*as* requires high temperature treatment. The in situ heating holder allows heating sample inside the TEM column (Supplementary Fig. 3), and removing guest molecules from MIL-53*as*. In our heating 3D ED experiments, the temperature was increased to 603 K from 298 K at a rate of 5 K/min and maintained at 603 K for 2 h before 3D ED data collection inside the high vacuum TEM column (<1×10^−5^ Pa) (MIL-53*as*-ht-v, Fig. 2e). As a result, the resolution of 3D ED data reached 0.81 Å and the completeness achieved 99.7%. The unit cell parameters were determined as *a* = 6.61(3) Å, *b* = 17.25(3) Å, *c* = 12.81(1) Å, *α* = *β* = *γ* = 90°, *V* = 1461(7) Å^3^. Ab initio structure solution of MIL-53*as*-ht-v (space group *Imma*) was obtained using direct methods (Table 1). There was no residual potential in the pores, indicating the full removal of the guest terephthalic molecules (Fig. 4e, Supplementary Figs. 24, 25b, and Supplementary Tables 10–12). After the temperature returned back to 298 K, 3D ED data were collected again and the obtained phase was named as MIL-53*as*-ht-vrt (rt: high temperature, Table 1 and Supplementary Fig. 26). Of note, compared with MIL-53*lt*-v which was also collected under same conditions of high vacuum and room temperature, 3D ED data of MIL-53*as*-ht-vrt (Supplementary Fig. 26 and Supplementary Tables 13–15) showed sharp diffraction spots without diffuse streaks. The slight diffuse streaks observed in the 3D ED data of MIL-53*lt*-v (Supplementary Fig. 9a–c) may come from the structure disorders due to the incomplete removal of guest molecules, so the full activation process before collecting data is very important for structure solution of flexible frameworks with empty channels. Moreover, in situ heating 3D ED experiments were also performed using in situ gas holder with MIL-53*lt*. 3D ED data were collected in the air (0.1 MPa) at high temperature (603 K), which gave a phase (named as MIL-53*lt*-ht-gas) (Table 1, Supplementary Fig. 27 and Supplementary Tables 16–18) similar to MIL-53*as*-ht-v with empty channels.

In this work, we demonstrate that atomic-level structural analysis of single microcrystals under various conditions is achievable by integrating 3D ED with environmental TEM. In addition to high vacuum condition at room or low temperature, heating, gaseous and liquid environments were also created with the help of in situ TEM holders. Ab initio structure solutions of microcrystals were successfully obtained using 3D ED under the high-temperature and gaseous environments. The determination of MIL-53 and one zeolite three-dimensional crystal structure in the liquid water was also achieved. The findings and experimental capability established here raise the prospect of future works on studying structural responsiveness to external stimuli, like gas, temperatures or even chemical processes.

## Methods

### Materials preparation

The preparation method of MIL-53*as* (Al) followed a previously reported literature^14^. In a typical procedure, 1.300 g Al(NO_3_)_3_·9H_2_O (Greagent, AR), 0.288 g terephthalic acid (H_2_BDC, J&K, > 99%) and 5 g distilled water were mixed in a 23 mL Teflon-lined autoclave. The reaction was kept at 493 K under autogenous pressure for three days. After being cooled down naturally, the white product was centrifuged and washed by distilled water for three times, which was MIL-53*as* at this stage. To evacuate extra H_2_BDC in the pores, the powder was treated at 603 K in the air for three days. After being exposed to the air at room temperature, the resulting light brown powder was MIL-53*lt*. Due to large particles size of MIL-53, sample was crushed in advance for 3DED experiments.

### Electron dose calculation

In the TEM JEM-F200 we used for experiments, there are two detectors: Cheetah 1800 from ASI (Amsterdam Scientific Instruments B.V.) and Rio-16 from Gatan, Inc. The counting efficiency of Rio-16 (1 count = 104 e^−^ in our case) was calibrated by the Gatan company before delivery. We can directly read the average electron dose using this camera with an empty area without sample and carbon film. Therefore, we used this Rio-16 camera as a reference to calculate the electron dose and the counting efficiency of Cheetah 1800. In particular, under very low electron dose, the signal to noise ratio in Rio-16 camera might be too low to have an accurate evaluation of electron dose. Instead, we used ASI detector (with a known counting efficiency calculated before) to calibrate the electron dose. It is worth mentioning that a more accurate method is to use Faraday cup to measure the beam current at the sample position. However, we have no access to this kind of facility now. The electron dose was then calculated using an indirect way.

### 3D ED experiments

The 3D ED datasets were collected on JEM-F200 TEM (JEOL Ltd. Model JEM-F200 with Schottky Gun, 200 kV) equipped with a hybrid-pixel electron detector (Cheetah 1800, 512 × 512 pixels, pixel size 55 μm, Amsterdam Scientific Instruments) and the software *Instamatic*^22^.

Cryogenic sample preparation of MIL-53*lt*-cryo and MIL-53*as*-cryo followed the plunge-freezing protocol, which was widely used in biological sample preparation^23^. This cryogenic sample preparation was also used in our previous report^24^. The crushed MIL-53 particles were dispersed in distilled water. After being sonicated for 10 min or without sonication (Supplementary Fig. 13), the solution was dropped onto a grid with ultrathin carbon film support (Beijing Zhongjingkeyi Technology Co., Ltd). Then the grid, which was fixed at the bottom of a plunger, was dropped into the liquid ethane, and was then transferred to a storage box in liquid nitrogen. Next, the cryogenic holder (Gatan, Inc., Model 914, Supplementary Fig. 1) was inserted into the cryogenic workstation and covered with liquid nitrogen. Later, the storage box with the grid was transferred into the workstation and the grid was loaded onto the cryo-transfer holder. Finally, the holder was inserted into the TEM for 3D ED experiments.

In a control experiment, the grid with MIL-53 particles was simply loaded onto the holder without procedures of plunge-freezing. The holder was fully inserted into TEM and then cooled down with liquid nitrogen (MIL-53*lt*-v-cryo: Supplementary Fig. 9d–f, MIL-53*as*-v-cryo: Supplementary Fig. 23c).

For cryogenic 3D ED experiments, the used electron dose rate was around 0.045 e^−^Å^−2^s^−1^. The high tilting cryo-transfer holder (Gatan, Inc., Model 914, Supplementary Fig. 1) was used to collect data. Each single dataset covered a tilt range of about 90° to 110°.

For high-temperature experiments (MIL-53*as*-ht-v and MIL-53*as*-ht-vrt), MIL-53*as* particles were dispersed with distilled water before being loaded on the in situ heating chips (Protochips Inc., Fusion Select Heating, model E-FHDC-VO, holey carbon film with the thickness of around 18 nm). After the sample was dried in the air, the in situ heating holder (Protochips Inc., Fusion Select holder, Supplementary Fig. 3) with the chip was inserted into the JEM-F200 TEM and treated with heating rate of 5 K/min to 603 K and kept for 2 h before being exposed to the electron beam (200 kV, around 0.01 e^−^Å^−2^s^−1^). 3DED data were also collected after the temperature returned to 298 K (MIL-53*as*-ht-vrt, kept at 298 K for 10 min before data collection, around 0.045 e^−^Å^−2^s^−1^). Each single dataset covered a tilt range of about 45° to avoid the collision of in situ holders with the pole piece of TEM.

For gas 3D ED experiments (MIL-53*lt*-gas (air)), MIL-53*lt* particles were dispersed in distilled water before being loaded on the bottom chip (Protochips Inc., EAB-33AB, thickness of window: 30 nm, thickness of spacer: 5 μm). After the solution was vapored in the air, the bottom chip was assembled with a top chip (EAT-33W, thickness of window: 50 nm) to form a closed gas cell on the top of in situ gas holder (Protochips Inc., Atmosphere holder, Supplementary Fig. 2), which contained static air (0.1 MPa). Then the leak check procedure was conducted to confirm a proper sealing of the gas cell and the existence of the air. The beam dose rate on the JEM-F200 TEM was reduced to around 0.045 e^−^Å^−2^s^−1^. Besides, for gas-heating 3D ED experiments (MIL-53*lt*-ht-gas), the sample was heated with a rate of 30 K/min to 603 K and kept for 10 min before being exposed to the electron beam for data collection (200 kV, around 0.01 e^−^Å^−2^s^−1^). Each single dataset covered a tilt range of about 45°.

For liquid 3D ED experiments (MIL-53*lt*-liquid (H_2_O)), the sample was dispersed with distilled water before being loaded on the in situ top chip (Protochips Inc., EPT-55W, thickness of window: 50 nm). When the top chip was dried, it was assembled with a bottom chip (EPB-55MF, thickness of spacer: 5 μm) with 0.1 μL distilled water on the window to form a closed liquid cell. The cell was loaded on the top of an in situ liquid holder (Protochips Inc., Poseidon holder, Supplementary Fig. 2, similar to Atmosphere holder). In the liquid 3D ED experiment of MIL-53, static mode of liquid was sealed inside the liquid cell instead of flowing mode. The leak check procedure was conducted to confirm the proper sealing of the liquid cell. The beam dose rate on the microscope JEM-F200 was reduced to around 0.045 e^−^Å^−2^s^−1^. Then the beam shower was performed in low magnification mode (×50) for 30–60 min to make the liquid layer thinner. Again, each single dataset covered a tilt range of about 45°.

The schematic of using environmental 3D electron diffraction in studying MIL-53 microcrystals was shown in Fig. 1b. In the initial stage of liquid 3D ED experiments, we met many problems in the data collection. The biggest issue was that the thick liquid layer would bring terrible backgrounds. Particles could not be distinguished from the liquid layer background. And no reflections of the particle could be observed, even the central spot.

A practical way to make the liquid layer thinner in the cell was using e-beam shower^25^. After 30–60 min beam shower under low mag conditions (×50), the liquid layer was scattered away by the gas bubbles generated by water decomposition under electron beam irradiation (Supplementary Fig. 16). As a result, the better contrast was achieved to locate crystals. Then a particle covered with a thin liquid layer is selected, from which SAED pattern taken along near the [001] axis showed a good signal-to-noise ratio (Supplementary Fig. 17). However, when we changed to mag mode (×15 K) from low mag mode (×50), the electron dose increased and unexpected liquid droplets aggregation appeared (Supplementary Movie 1), which led to a worse background. This phenomenon might come from the charging of chips under electron irradiation.

During the electron beam irradiation, bubbles would generate and induce the flow of liquid. The movement of liquid would further led to the drift of particles. Moreover, if a particle was suspended in the liquid, random rotation would occur during the beam irradiation. As shown in the Supplementary Movie 2 (random rotation of a MIL-53 particle suspended in water layer), the ED patterns changed automatically without tilting the holder. To avoid these effects, sample was dried and stabilized on the top chip before introducing liquid water environment.

The feasibility of liquid 3D ED was further proved using LTA zeolite immersed into liquid water, from which the 3D ED data were collected and used to successfully obtain the ab initio structure solution (Supplementary Figs. 19 and 20).

### Scanning electron microscopy

The morphology of MIL-53 products was investigated using a field-emission scanning electron microscope (JSM-7800F Prime, 1 kV, working distance: 8.0 mm, emission current: 43 μA, Supplementary Figs. 4 and 5). It’s worth mentioning that the vacuum value was below 4.4 × 10^−4^ Pa in the SEM, which might induce the escape of guest H_2_O inside the channels and influence the real morphology of MIL-53*lt*.

### Powder X-ray diffraction data

All the data were collected on a Bruker D8 Advance Diffractometer with the 2*θ* range of 5–30° (40 kV, 40 mA, *λ* = 1.5418 Å). For MIL-53*as* and MIL-53*lt*, PXRD data were obtained at ambient temperature (298 K) and pressure (0.1 MPa). Supplementary Fig. 6 is the data from MIL-53*as*.

For MIL-53*lt*, in order to verify the feasibility of gas 3D ED and liquid 3D ED, the following PXRD experiments are conducted: one sample was exposed to the air (Supplementary Fig. 7, red curve), the other one was immersed into distilled water (Supplementary Fig. 7, blue curve). Both results showed good match with the simulated PXRD pattern of MIL-53*lt*.

### In situ PXRD data

The in situ PXRD experiments indicated the flexibility of MIL-53 framework and the phase transitions under different conditions, which gave guidance to environmental 3D ED experiments. 0.1 g MIL-53 powder was loaded onto an in situ experiment module. The initial state of the powder (Supplementary Fig. 8, blue curve) matched well with MIL-53*lt* (Supplementary Fig. 8, black curve). Then atmospheric pressure was pumped to 80 Pa (the lowest pressure of the equipment), and no apparent phenomenon was observed (Supplementary Fig. 8, green curve). After being heated to 373 K for 8 min, and kept at 373 K for 4 min, the phase turned to be MIL-53*ht* phase (Supplementary Fig. 8, purple curve, compared with red curve). When the temperature was reduced to 298 K, the phase (Supplementary Fig. 8, brown curve) changed back to MIL-53*lt* (the pressure is still 80 Pa). The final state matched with MIL-53*lt* phase after completely back to initial state (298 K, 0.1 MPa). The in situ PXRD experiments showed the reversible “breathing effect” of MIL-53. The differences between in situ PXRD results and 3D ED results of MIL-53*lt*-v are due to different magnitude of pressures that 80 Pa for PXRD and around 10^−5^ Pa in the TEM column.

### Thermogravimetry analysis

TG experiments were performed as MIL-53*as* in the oxygen flow (20.0 ml/min) from 303 K to 973 K (5 K/min) on a PerkinElmer TGA 8000. The result showed that the guest terephthalic /framework terephthalic ratio is around 0.55/1 (Supplementary Fig. 22).

### Structure solution

3D ED Dada except for MIL-53*lt*-liquid (H_2_O) were processed with XDS software^26^ to get unit cell parameters and reflection intensity information while MIL-53*lt*-liquid (H_2_O) were processed by the software EDT-Process. Ab initio structure solutions of all phases were obtained using direct methods implemented in SIR2014 software^27^, and electron atomic scattering factors^28^ were used in structure solution and refinement. All projections and slices of 3D ED data (See Supplementary Figures) were processed by the software EDT-Process.

### Data merge strategy

Limited by the low completeness of each single dataset, ab initio structure solution was not successful in some cases. Merging multiple datasets can improve data quality. The datasets processed with XDS were merged with XSCALE program^29^.

### Structure refinement against 3DED data

The structure refinements were performed using SHELXL program^30^ in Olex2 packages^31^ by assuming kinematical scattering (See Supplementary Tables). Some framework atoms were missing in the initial structure solutions obtained from SIR2014 software^27^ and all of them were successfully found during the following refinement process. Anisotropic refinement was initially introduced for all structures. However, negative ADPs values of atoms in MIL-53*lt*-gas and MIL-53*lt*-cryo were found which indicated some unreasonable structural refinements. Therefore, these two structures were refined with isotropic ADPs while other structures were generally completed with anisotropic ADPs. Since the residual electrostatic potential in the channels of MIL-53*as*-v was found, some C atoms were added as guest molecules and all of them were refined with isotropic ADPs. Moreover, to have a reasonable structure, SHELX instructions such as FLAT, SADI, DFIX, SIMU and DELU were used to restrain the structure model during the refinements.

## Supplementary information

Supplementary Information

Description of Additional Supplementary Files

Supplementary Movie 1

Supplementary Movie 2

## Data Availability

Additional illustrations of environmental 3D ED experiments, scanning electron microscopy, powder X-ray diffraction, thermogravimetry analysis, 3D electron diffraction data, liquid TEM experiments and tables of crystallographic information are available in the Supplementary Information or Supplementary Movies. Crystallographic data for 3DED-collected MIL-53*lt*-cryo, MIL-53*lt*-gas (air), MIL-53*lt*-liquid (H_2_O), MIL-53*as*-v, MIL-53*as*-ht-v, MIL-53*as*-ht-vrt and MIL-53*lt*-ht-gas structures are available free of charge via the Cambridge Crystallographic Data Centre (CCDC), with deposition numbers. 2124556 [10.5517/ccdc.csd.cc299s2h], 2124554 [10.5517/ccdc.csd.cc299s0f], 2124553 [10.5517/ccdc.csd.cc299rzc], 2124555 [10.5517/ccdc.csd.cc299s1g], 2168111 [10.5517/ccdc.csd.cc2bs32b], 2167321 [10.5517/ccdc.csd.cc2br8lz] and 2167322 [10.5517/ccdc.csd.cc2br8m0], respectively. Source data are provided with this paper.

## Source data

Source Data
