# Supplementary material for: Atomic-level structural responsiveness to environmental conditions from 3D electron diffraction
Source: Nat Commun. 2022 Nov 4;13:6625. doi: 10.1038/s41467-022-34237-1 (PMC9636419; doi:10.1038/s41467-022-34237-1)
Supplement: Supplementary file 1 — Supplementary Information [file 41467_2022_34237_MOESM1_ESM.pdf]

Supplementary Information

Atomic-level structural responsiveness to environmental conditions from 3D electron diffraction

Yang Ling *et al*

Section 1 | Supplementary Figures

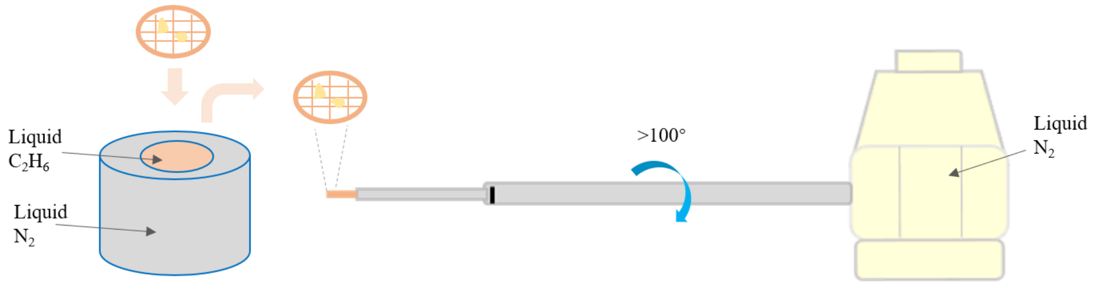

Supplementary Figure 1 | The schematic representation of cryogenic 3D ED experiments. The grid with the sample, which is fixed at the bottom of a plunger, is dropped into the liquid ethane, and is then transferred to a storage box in liquid nitrogen. Next, the cryogenic holder is inserted into the cryogenic workstation and covered with liquid nitrogen. Later, the storage box with the grid is transferred into the workstation and the grid is loaded onto the cryo-transfer holder. Finally, the holder is inserted into the TEM for cryogenic 3D ED experiments. The cryo-transfer holder can be tilted around ±50°.

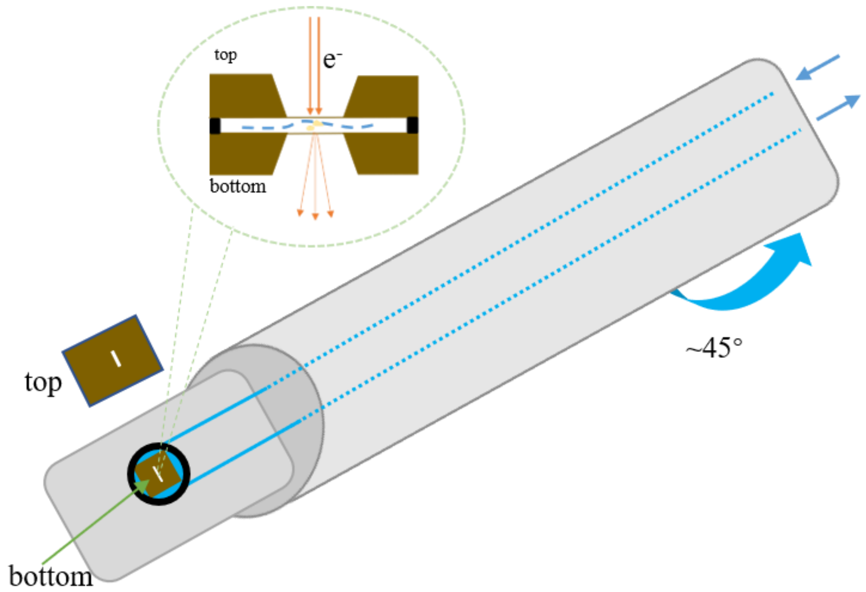

Supplementary Figure 2 | The schematic representation of *in situ* gas or liquid 3D ED experiments. The *in situ* gas/liquid cell is composed of two Micro-Electron-Mechanical-System (MEMS, an integrated circuit for heating) based chips and an 'O' ring for sealing. The gas/liquid can flow through the inlet and outlet microfluidic pipelines as shown with blue lines and arrows. The maximum tilting angle is limited within 45°.

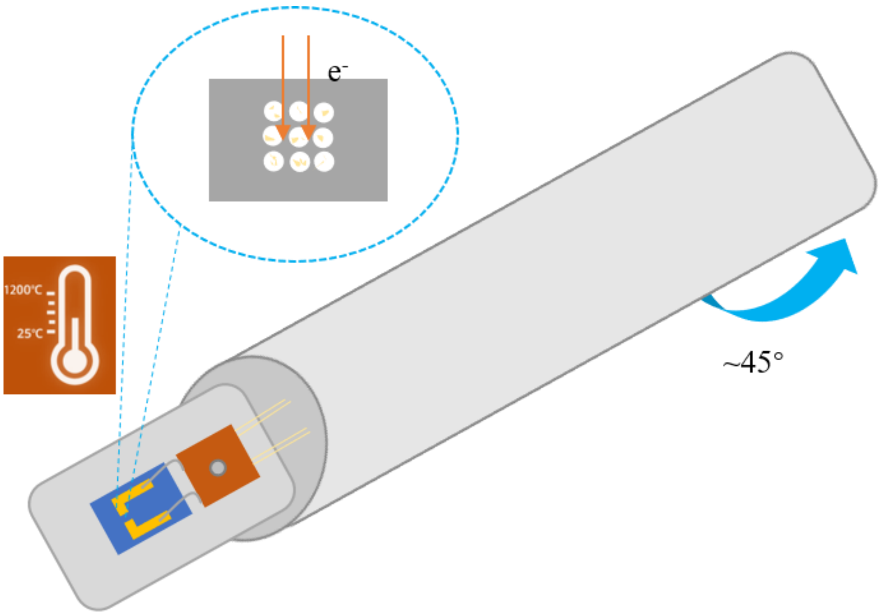

Supplementary Figure 3 | The schematic representation of *in situ* heating 3D ED experiments. The heating inside TEM column environment is enabled by a single MEMS chip with an integrated circuit. There are 9 observation areas on the *in situ* heating chip. The temperature can be controlled by with electric current to the circuit from 298 K to 1473 K. The maximum tilting angle is limited to 45°.

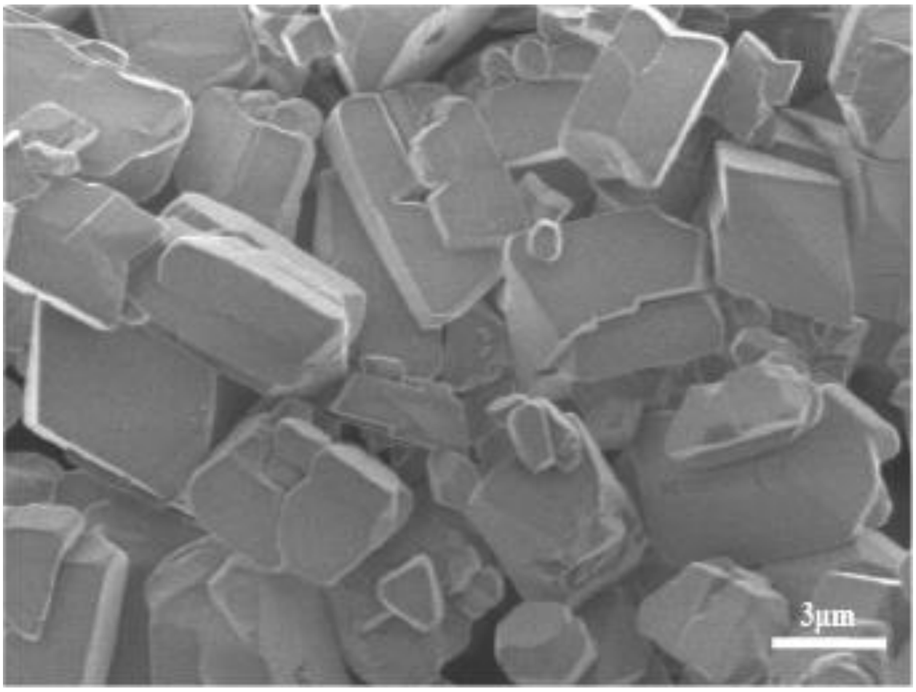

Supplementary Figure 4 | SEM image of particles prepared with MIL-53*as* powder (JSM-7800F Prime, accelerating voltage: 1 kV, working distance: 8.0 mm, emission current: 43 μA, 4.4×10^{-4} Pa).

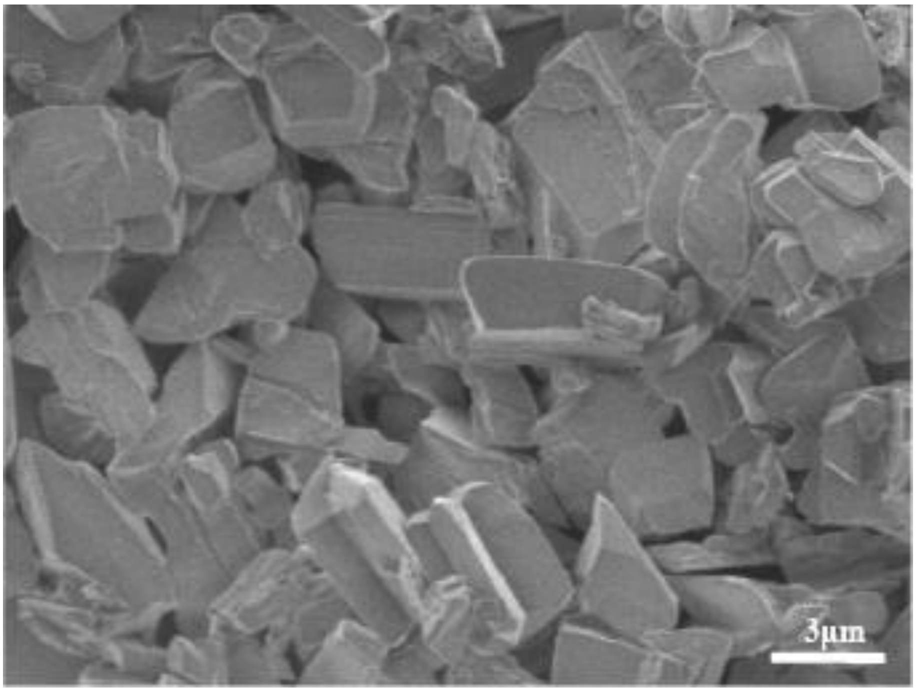

Supplementary Figure 5 | SEM image of particles prepared with MIL-53*lt* powder (JSM-7800F Prime, accelerating voltage: 1 kV, working distance: 8.0 mm, emission current: 43 μA, 4.4×10^{-4} Pa). It's worth mentioning that the vacuum value was below 4.4×10^{-4} Pa in the SEM, which might induce the escape of guest water inside the channels and influence the real morphology of MIL-53*lt*.

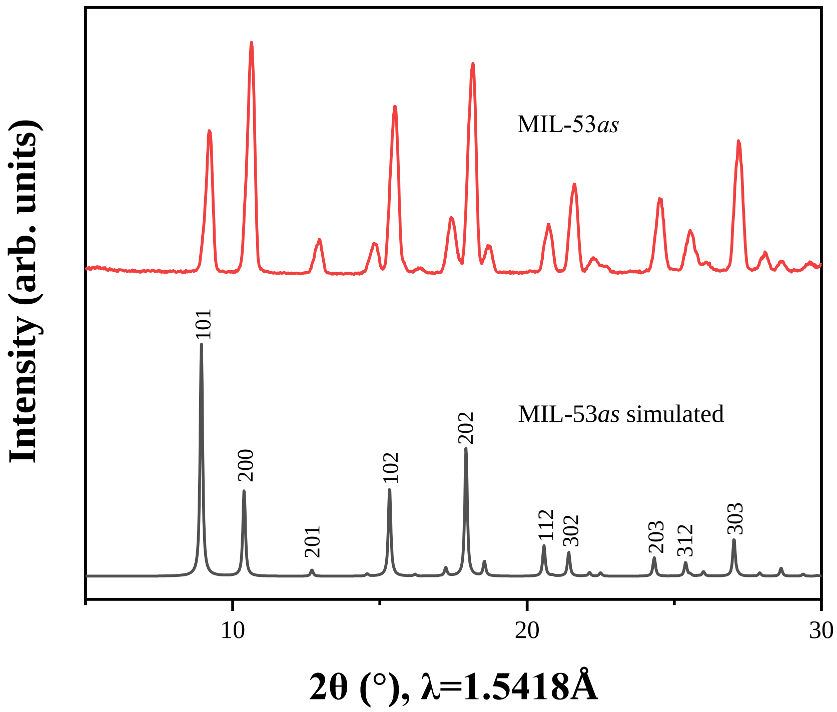

Supplementary Figure 6 | PXRD data of MIL-53*as*. Data were collected at ambient temperature (298 K) and pressure (0.1 MPa) on a Bruker D8 Advance Diffractometer with the 2θ range of 5-30° (40 kV, 40 mA, λ = 1.5418 Å). The observed profile (red) matches with the simulated PXRD one (black).

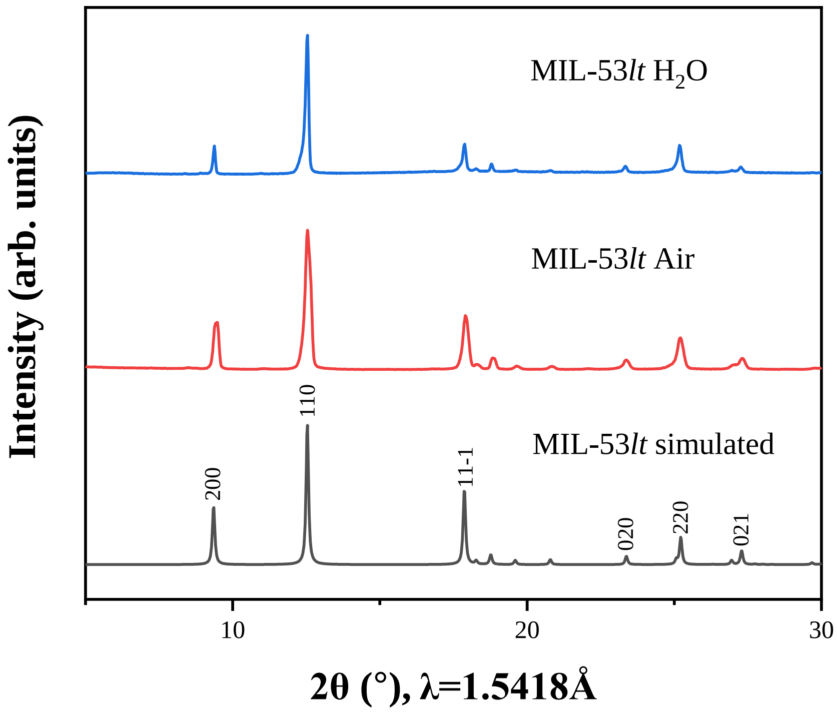

Supplementary Figure 7 | PXRD data of MIL-53*lt*. Data were collected at ambient temperature (298 K) and pressure (0.1 MPa) on a Bruker D8 Advance Diffractometer with the 2θ range of 5-30° (40 kV, 40 mA, λ = 1.5418 Å). In order to verify the feasibility of gas 3D ED and liquid 3D ED, the PXRD experiments were conducted: one was exposed to the air (red), the other sample was immersed into distilled water (blue). Two PXRD patterns are very similar and show a good match with the simulated PXRD pattern of MIL-53*lt* (black).

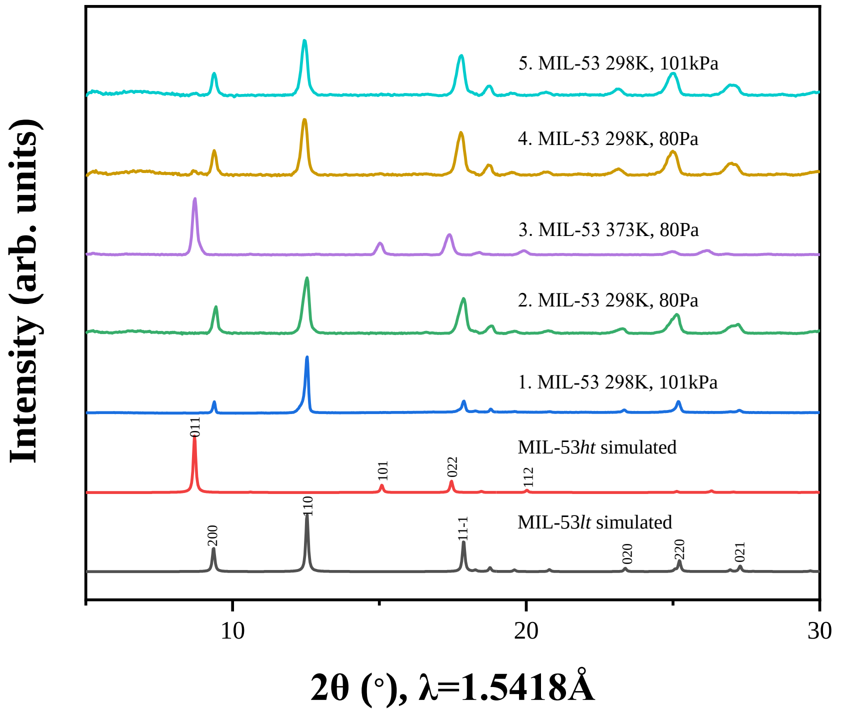

Supplementary Figure 8 | *In situ* PXRD data of MIL-53. Data were collected on a Bruker D8 Advance Diffractometer with the 2θ range of 5-30° (40 kV, 40 mA, λ = 1.5418 Å). The *in situ* PXRD experiment show the reversible "breathing effect" of MIL-53. 0.1g MIL-53 powder was loaded onto an *in situ* experiment module. The initial state of the powder (298 K, 0.1 MPa, blue) matched well with simulated PXRD of MIL-53*lt* (black). Then atmospheric pressure was pumped to 80 Pa (the lowest pressure of the equipment), and no apparent phenomenon was observed (green). After being heated to 373 K, the phase turned to be MIL-53*ht* phase (purple curve, matched with red). When the temperature was reduced to 298 K, the phase (brown) changed back to MIL-53*lt* (the pressure was still 80 Pa). The final state matched with MIL-53*lt* phase after completely back to the initial state (298 K, 0.1 MPa, cyan).

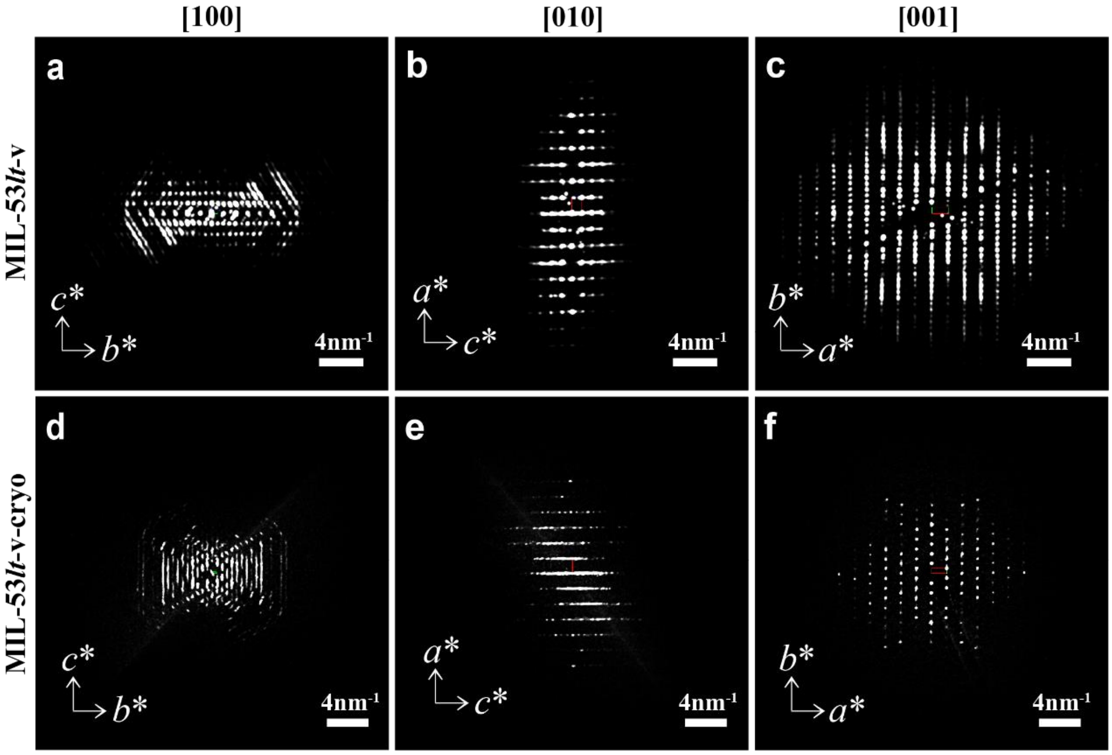

Supplementary Figure 9 | The projections of 3D ED data from (a-c) MIL-53*lt*-v (298 K) and MIL-53*lt*-v-cryo (only frozen by liquid nitrogen but without the plunge-freezing and cryogenic transfer protocols) (d-f), respectively.

For MIL-53*lt*-v, the observed reflection conditions are summarized as *hkl: h* + *k* + *l* = 2*n*; *hk*0*: h*, *k* = 2*n*; *h*0*l: h* + *l* = 2*n*; 0*kl: k* + *l* = 2*n*, which suggest two possible space groups: *Imma* (no. 74) and *Im*2*a* (no. 46). The space group *Imma* with *a* = 6.69(3) Å, *b* = 17.11(4) Å and *c* = 12.21(4) Å gave the best results of *ab initio* structure solution using direct methods. Slight diffuse streaks along c^* might indicate the existence of irregular stacking in the structure caused by imperfect guest H_2O removal.

For MIL-53*lt*-v-cryo, sample was prepared by inserting the holder with MIL-53*lt* into high-vacuum TEM column, followed by cooling down using liquid nitrogen. Unit cell parameters *a* = 6.62 Å and *b* = 20.54 Å are obtained. Compared with MIL-53*lt*-v, the obvious change of cell parameter *b* of MIL-53*lt*-v-cryo may be caused by the temperature-induced transition from large pores to low-temperature narrow pores. The diffuse streaks become more serious after this slow cooling process.

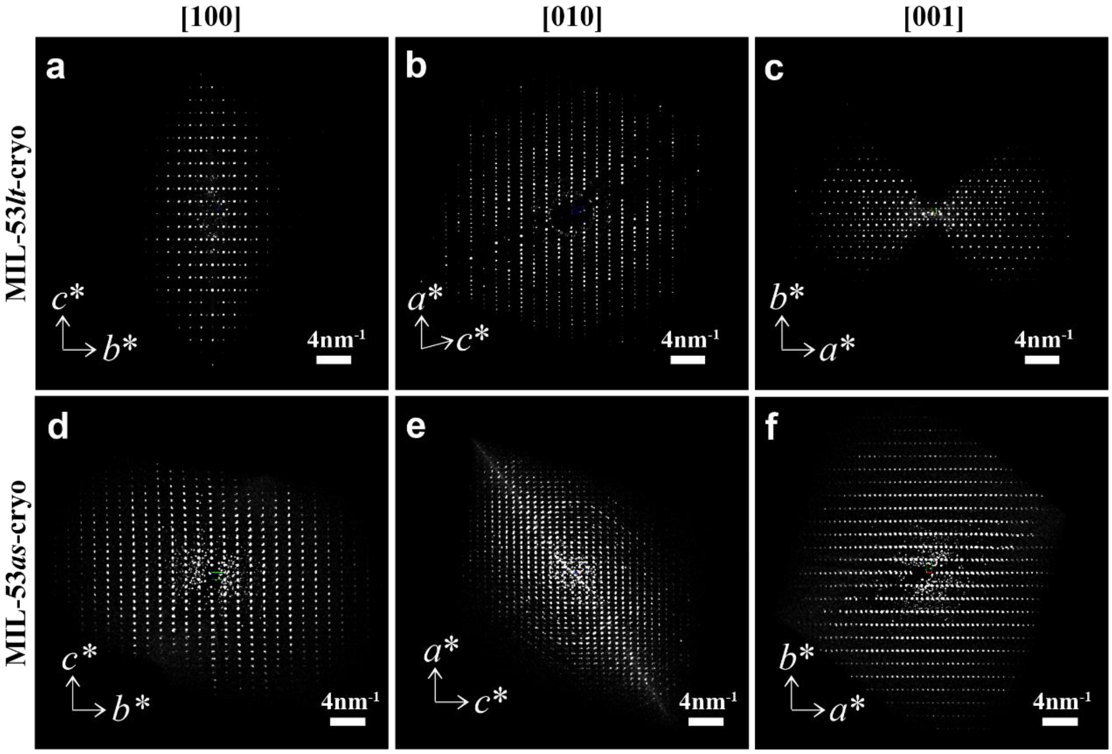

Supplementary Figure 10 | The projections of 3D ED data from (a-c) MIL-53*lt*-cryo and (d-f) MIL-53*as*-cryo (both prepared through the plunge-freezing and cryogenic transfer protocols) along (a-d) [100], (b-e) [010] and (c-f) [001] directions respectively.

The observed reflection conditions of MIL-53*as*-cryo are summarized as $0kl$: $k + l = 2n$; $hk0$: $h = 2n$, which suggest two possible space groups: *Pnma* (no. 62) and $Pn2_1a$ (no.33). Using direct methods, *ab initio* structure solution of MIL-53*as*-cryo is obtained with the space group *Pnma* with a = 17.12(5) Å, b = 6.55(6) Å and c = 11.87(2) Å.

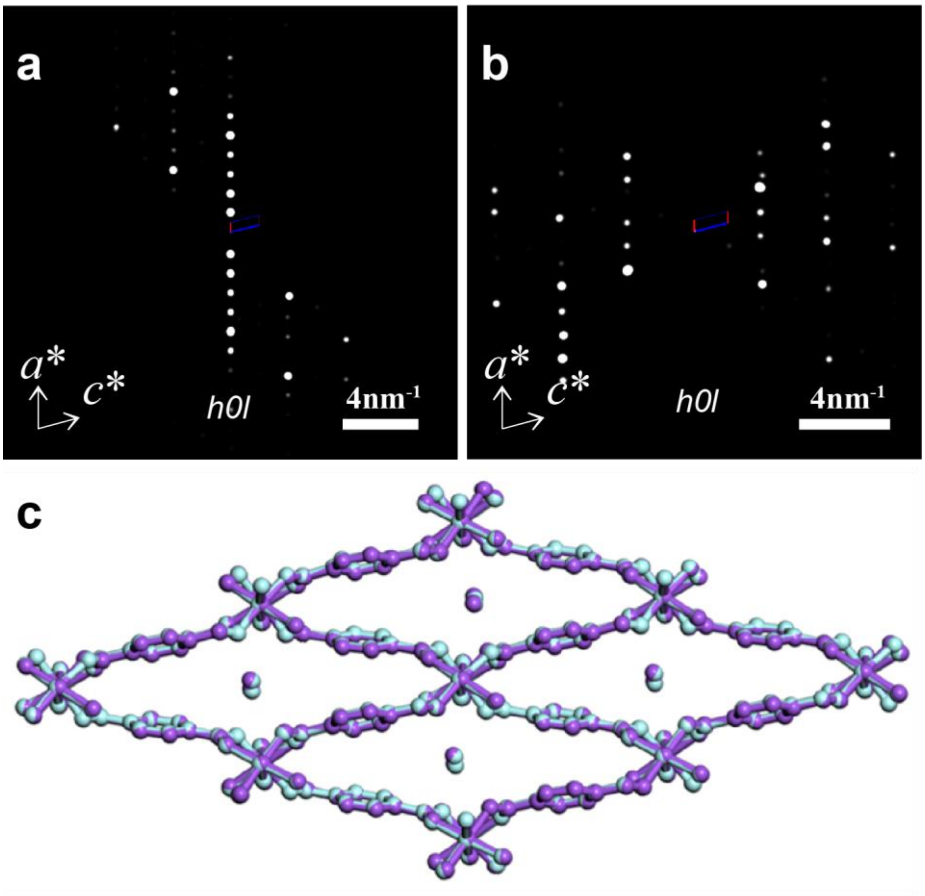

Supplementary Figure 11 | The slices at *h*0*l* cut from reconstructed 3D ED data of (a) MIL-53*lt*-cryo and (b) MIL-53*lt*-gas (air). (c) Structure solutions of MIL53lt-cryo using space group *P*2_1/*n* (purple) and *P*1 (cyan) are overlaid.

For MIL-53*lt*-cryo, the Laue class is determined as 1 2/*m* 1 with unique axis *b*. Reflection conditions are: for general *hkl*: $h \neq 0$, $k \neq 0$, $l \neq 0$ and for *h*0*l*: *h*, *l* = 2n. The allowed highest space group is *P*2_1/*n* (no. 14). To check the possibility of overestimating the space group symmetry, space group *P*1 (No. 1) is tried to obtain *ab initio* structure solution of MIL-53*lt*-cryo with direct methods, which matches well with that obtained using the space group *P*2_1/*n*. For MIL-53*lt*-gas (air), the observed reflection conditions are summarized as *hkl*: $h + k = 2n$ (Supplementary Fig. 13c) and *h0l*: *h*, $l = 2n$, which suggest two possible space groups: *Cc* (no. 9) and *C*2/*c* (no. 15). *Ab initio* structure solution of MIL-53*lt*-gas (air) is obtained with the space group *Cc* using direct methods.

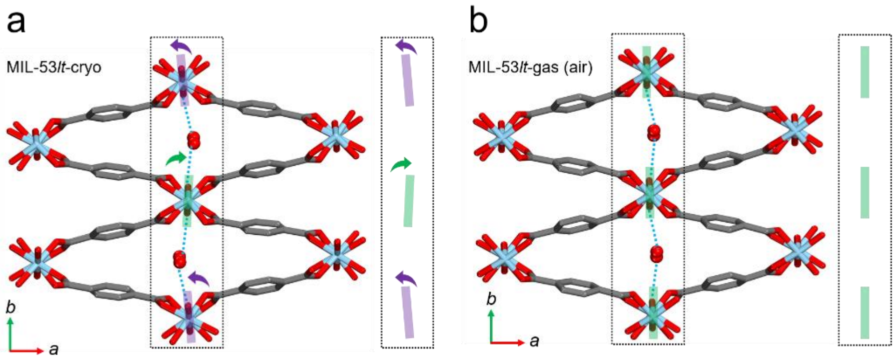

Supplementary Fig. 12 | Illustration of orientation of [AlO_6] octahedra in two structures (a) MIL-53*lt*-cryo and (b) MIL-53*lt*-gas (air), respectively.

The orientations of adjacent [AlO_6] octahedra along *b* axis are different in two structures, as illustrated in the dashed boxes. It is highly possible that hydrogen bonds exist between guest water molecules and oxygen atoms in the framework.

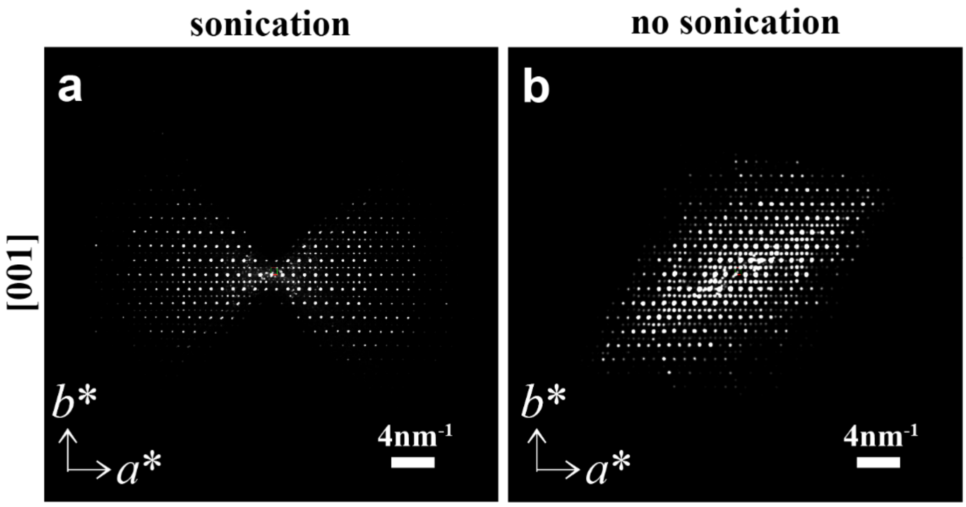

Supplementary Figure 13 | 3D ED data collected from MIL-53*lt*-cryo crystals using cryogenic sample preparation (a) with and (b) without ultrasound sonication before the cryogenic sample preparation. Extra reflections are observed in both cases.

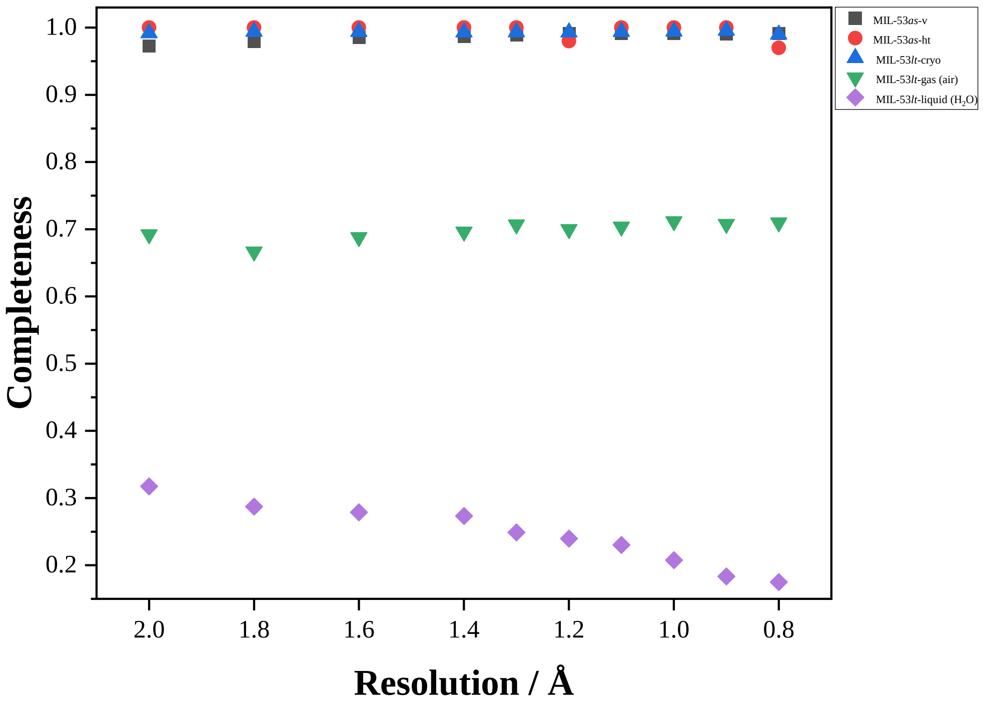

Supplementary Figure 14 | Relationships between the completeness and resolution of 3D ED data for different phases.
Due to different orientations of MIL-53 nanoparticles, merging of multiple datasets can improve the completeness. For MIL-53*as*-v, MIL-53*as*-ht-v and MIL-53*lt*-cryo, the completeness can reach 99%. However, the orientational effect become a serious factor for limited tilting-range *in situ* gas holders. The completeness of 3D ED data from MIL-53*lt*-gas (air) can only achieve around 70% even after merging several datasets.

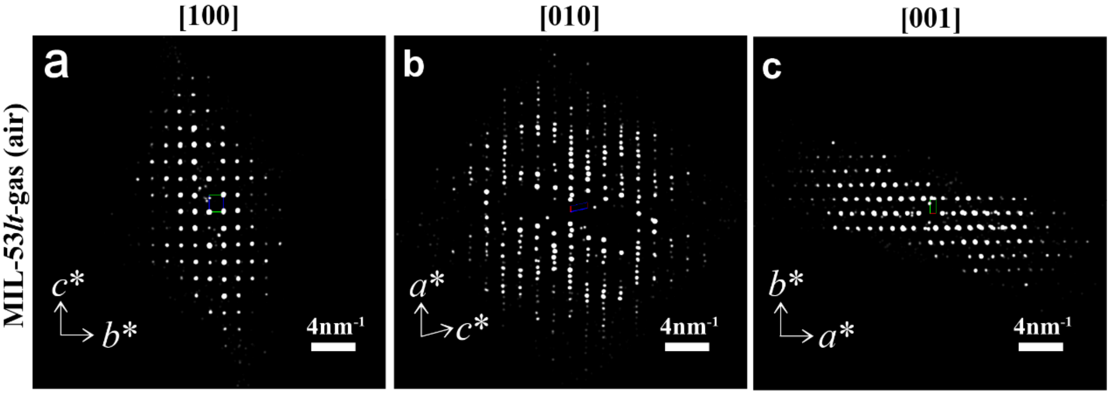

Supplementary Figure 15 | The projections of 3D ED data from MIL-53*lt*-gas (air) in the static air (0.1 MPa) along (a) [100], (b) [010] and (c) [001] directions respectively.

For MIL53*lt*-gas (air), the observed reflection conditions are summarized as *hkl*: $h + k = 2n$; $h0l$: $h, l = 2n$, which suggest two possible space groups: *C*2/*c* (no. 15) and *Cc* (no. 9). Using direct methods, *ab initio* structure solution of MIL-53*lt*-gas (air) is obtained with the space group *Cc* with a = 19.55(2) Å, b = 7.81(2) Å, c = 6.62(2) Å and β = 104.5(2)°

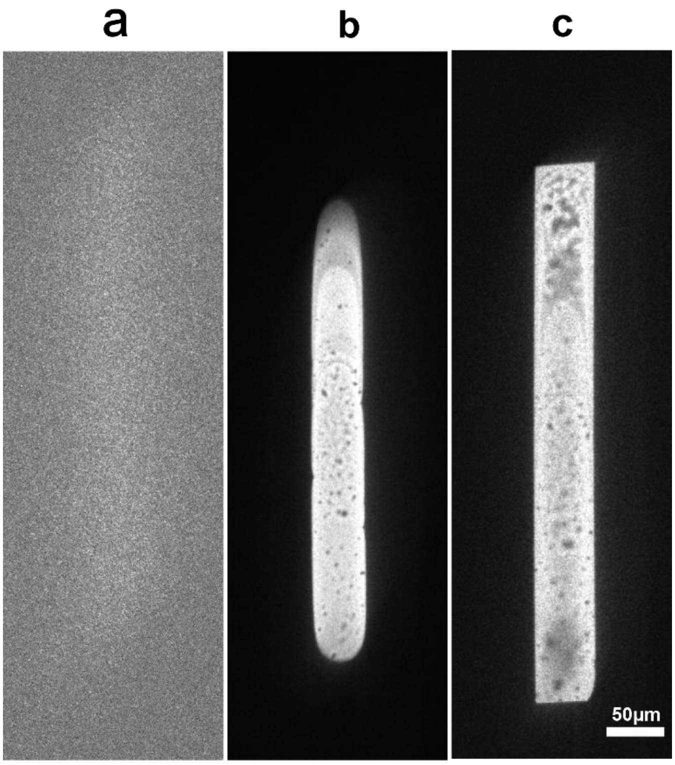

Supplementary Figure 16 | The beam shower process of the liquid layer. After the water was scattered away, the window of liquid cell could be clearly seen.

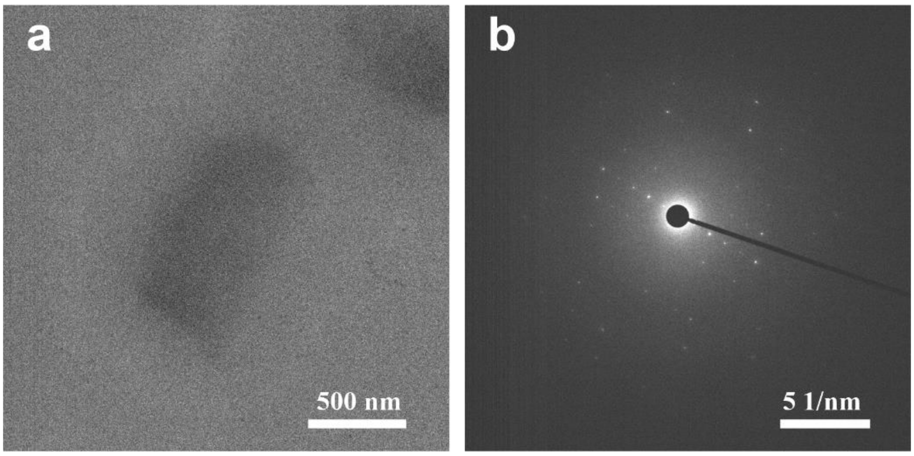

Supplementary Figure 17 | (a) The TEM image and (b) SAED pattern of the selected MIL-53*lt* particle covered with a thin layer of water.

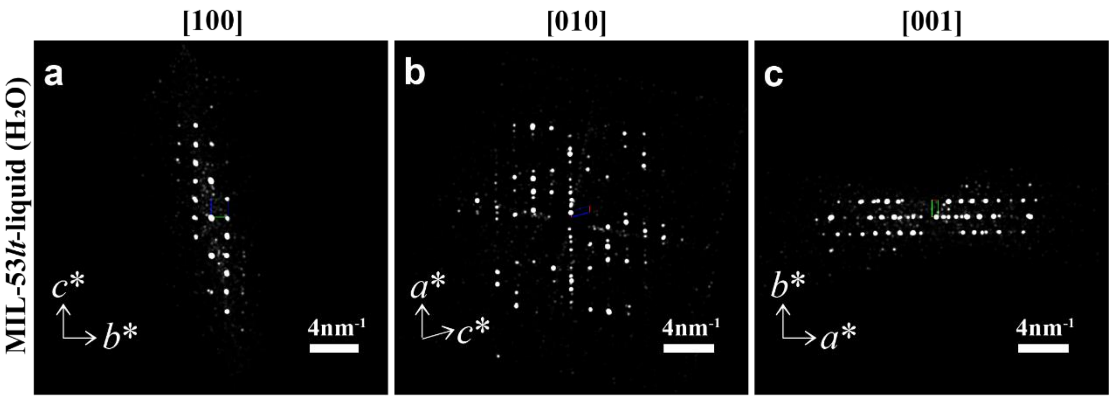

Supplementary Figure 18 | The projections of 3D ED data from MIL-53*lt*-liquid (H_2O) covered by the liquid layer along (a) [100], (b) [010] and (c) [001] directions respectively.

The reflection conditions cannot be summarized from experimental 3D ED data due to the low completeness. The space group is assumed to be same as MIL-53*lt*-gas (air). The cell parameters are a = 19.54 Å, b = 7.62 Å, c = 6.56 Å and β = 105.0°.

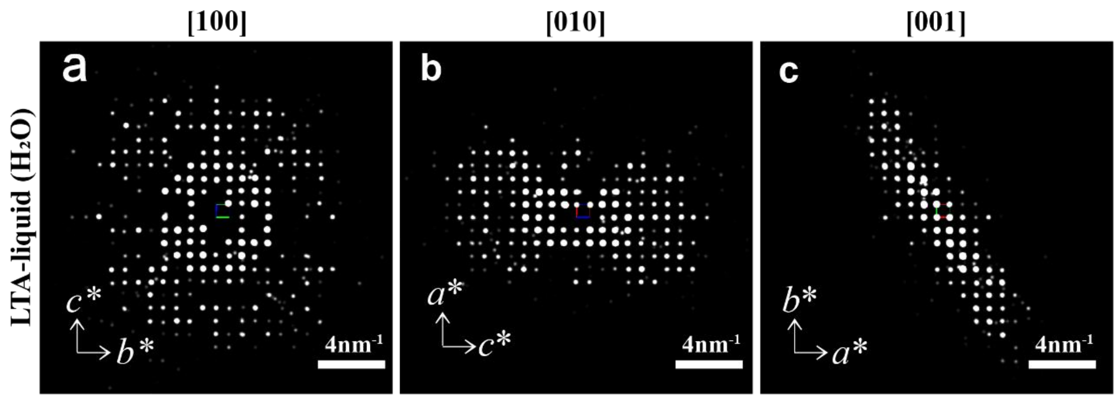

Supplementary Figure 19 | The projections of 3D ED data from LTA in the liquid water along (a) [100], (b) [010] and (c) [001] directions respectively.

For LTA in the liquid water, the unit cell is determined as $a = b = c = 12.3$ Å, $\alpha = \beta = \gamma = 90°$. No reflection conditions are summarized, so the possible space groups are: $P23$ (no. 195), $Pm\bar{3}$ (no. 200), $P432$ (no. 207), $P\bar{4}3m$ (no. 215) and $Pm\bar{3}m$ (no. 221). Using direct methods, the best *ab initio* structure solution of LTA is obtained with the space group $Pm\bar{3}m$.

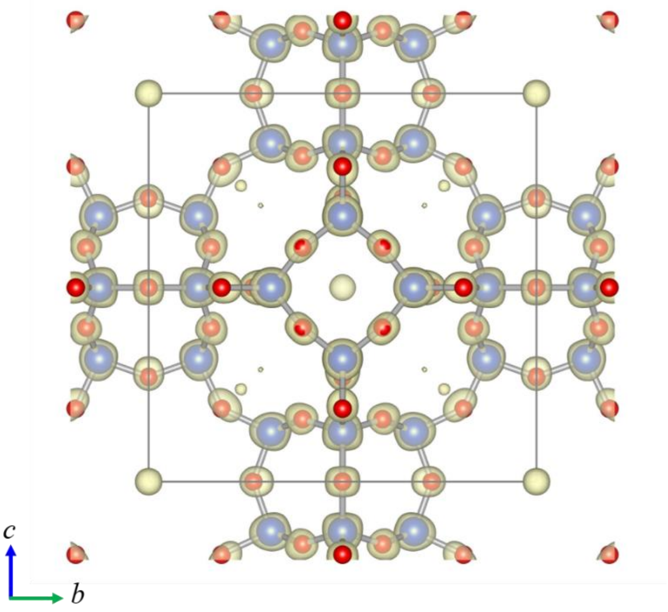

Supplementary Figure 20 | Electrostatic potential map overlaid with crystal structure model of LTA zeolite framework.

Blue: Si/Al atoms, red: O atoms. Here we use a cubic crystal system with the space group of *Pm*-3*m* and unit cell parameter of 12.3 Å without distinguishing Si from Al at each T position. The isosurface level is 2σ[*V*(r)].

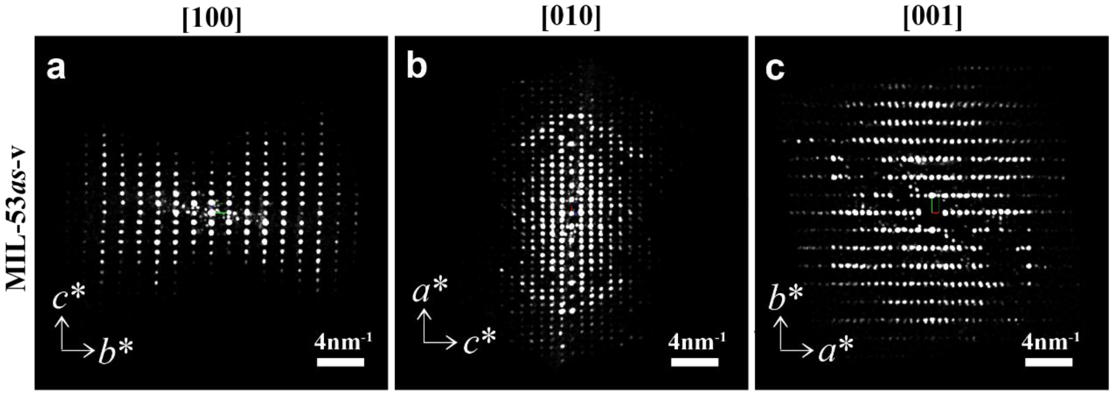

Supplementary Figure 21 | The projections of 3D ED data from MIL-53*as*-v (a-c) under the conditions of high vacuum and 298K along (a) [100], (b) [010] and (c) [001] direction respectively.

For MIL-53*as*-v, the observed reflection conditions are summarized as $0kl$: $k + l = 2n$; $hk0$: $h = 2n$, which suggest two possible space groups: *Pnma* (no. 62) and $Pn2_1a$ (no.33). Using direct methods, *ab initio* structure solution of MIL-53*as*-v is obtained with the space group *Pnma* with a = 17.03(1) Å, b = 6.57(2) Å and c = 12.16(2) Å.

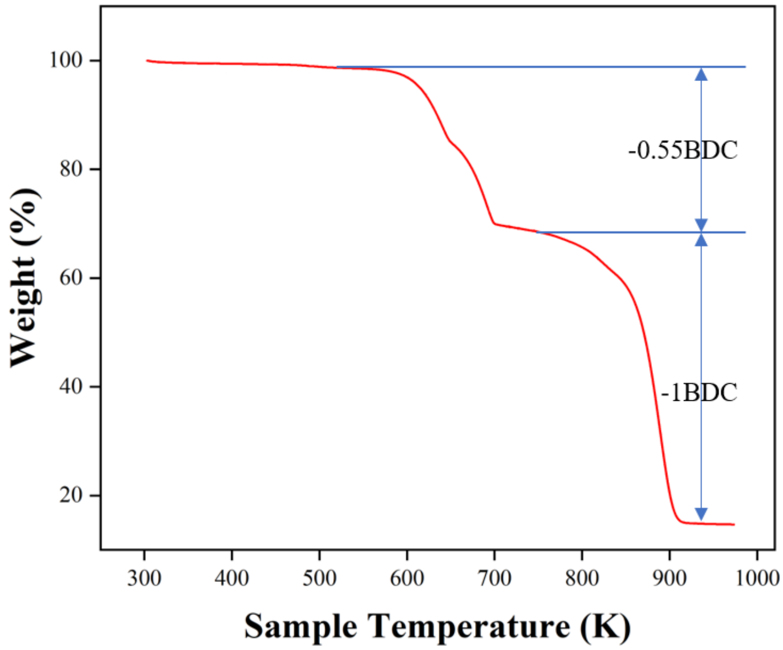

Supplementary Figure 22 | TG curves of MIL-53*as* (Al) in the oxygen flow (20.0 ml/min) from 303 K to 973 K (5 K/min).

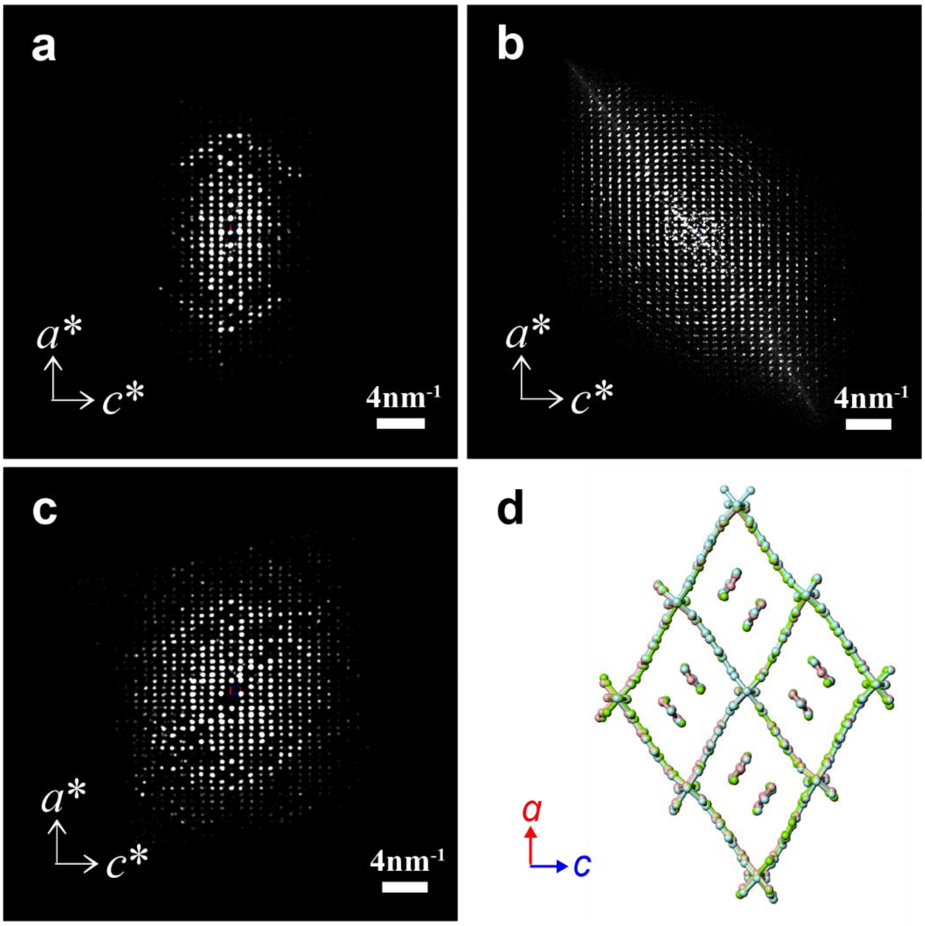

Supplementary Figure 23 | The projections of 3D ED data from (a) MIL-53*as*-v, (b) MIL-53*as*-cryo and (c) MIL-53*as*-v-cryo along [010] direction. (d) The three determined structures are overlaid (red: MIL-53*as*-v; green: MIL-53*as*-cryo; cyan: MIL-53*as*-v-cryo). MIL-53*as*-v: a = 17.03 Å, b = 6.57 Å and c = 12.16 Å. MIL-53*as*-cryo: a = 17.12 Å, b = 6.55 Å and c = 11.87 Å. MIL-53*as*-v-cryo: a = 17.41 Å, b = 6.69 Å and c = 12.09 Å. All these three structures are solved with the space group *Pnma*. Compared with 3D ED experiments of MIL-53*lt* (MIL-53*lt*-v, MIL-53*lt*-cryo and MIL-53*lt*-v-cryo), there are three main differences: (1) guest terephthalic acid molecules remained in the pore channels for MIL-53*as*-v, (2) no extra rows of reflections for MIL-53*as*-cryo and (3) no diffuse streaks for MIL-53*as*-v-cryo.

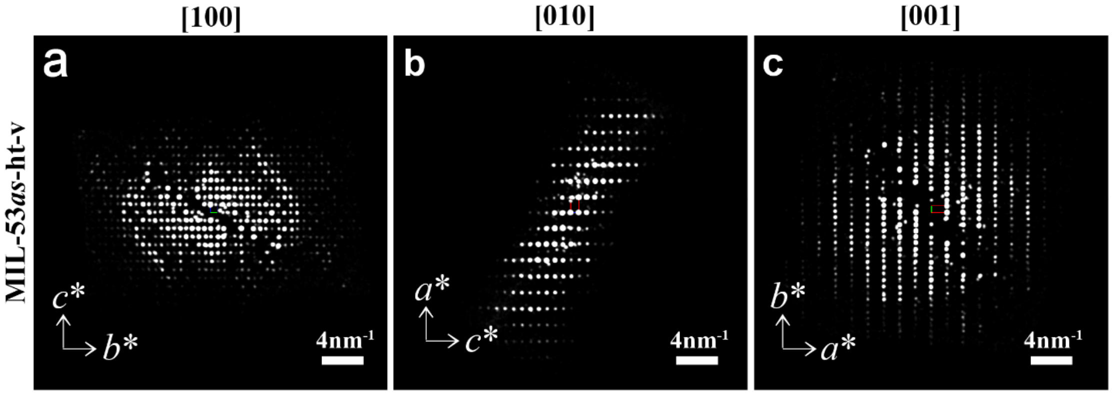

Supplementary Figure 24 | The projections of 3D ED data from MIL-53*as*-ht-v under the conditions of high vacuum and 603 K along (a) [100], (b) [010] and (c) [001] directions, respectively.

For MIL-53*as*-ht-v, the observed reflection conditions are summarized as *hkl: h* + *k* + *l* = 2*n*; 0*kl: k* + *l* = 2*n*; *h*0*l: h* + *l* = 2*n*. Some reflections appear in *hk*0 plane only satisfy *h* + *k* = 2n rather than *h*, *k* = 2n which suggest six possible space groups: *Immm* (no. 71), *Imm*2 (no. 44), *Im*2*m* (no. 44), *I*2*mm* (no. 44), $I2_12_12_1$ (no. 24) and *I*222 (no. 23). *Ab initio* structure solution of MIL-53*as*-ht-v is obtained by using direct methods with the space group $I2_12_12_1$. Finally, the software *Platon*[1] suggests the space group could be improved to *Imma* and the structure is further refined using the space group *Imma.*

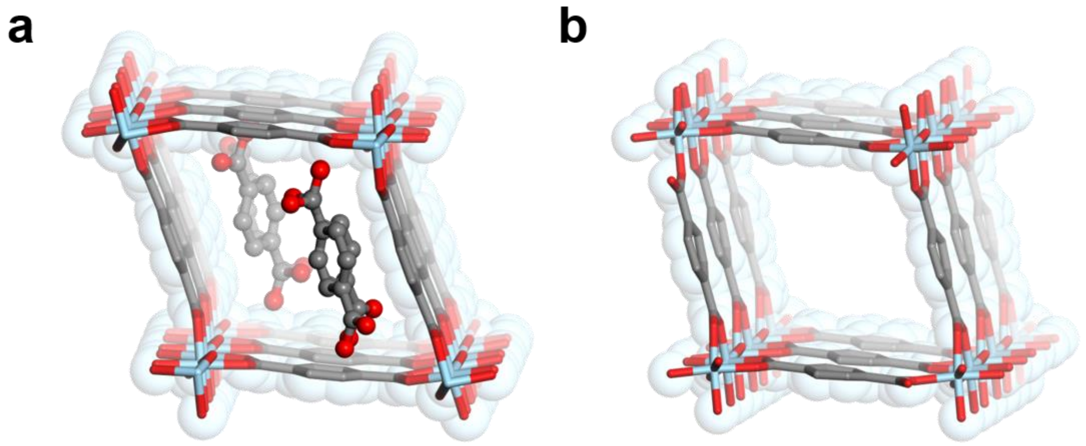

Supplementary Figure 25 | The schematic presentation of different phases from MIL-53*as* solved by environmental 3D ED.
(a) MIL-53*as*-v. **(b)** MIL-53*as*-ht-v. The space group changes from *Pnma* to *Imma* after heating treatment.

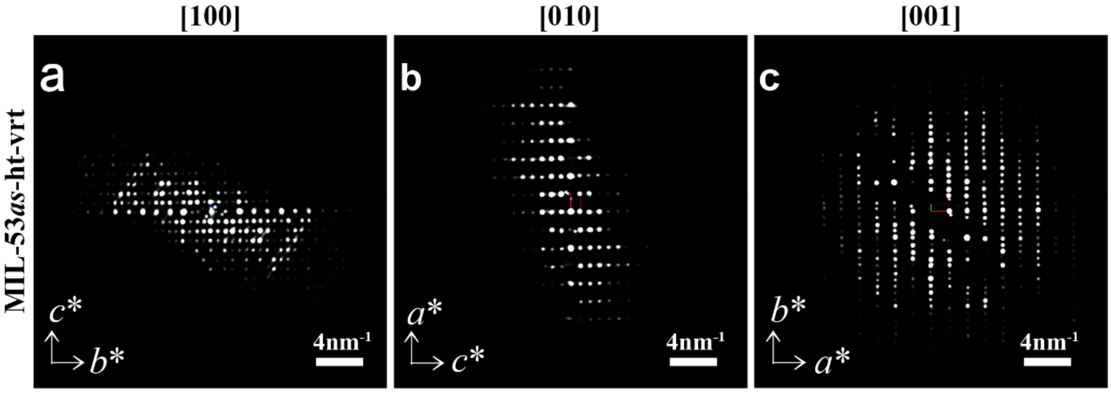

Supplementary Figure 26 | The projections of 3D ED data from MIL-53*as*-ht-vrt under the conditions of high vacuum and 298 K (after heating at 603 K) along (a) [100], (b) [010] and (c) [001] directions respectively.

For MIL-53*as*-ht-vrt, the observed reflection conditions are summarized as *hkl:* $h + k + l = 2n$; 0*kl:* $k + l = 2n$; *h*0*l:* $h + l = 2n$ and *hk*0: h , k = 2n, which suggest two possible space groups: *Imma* (no. 74) and *Im*2*a* (no. 46). Using direct methods, *ab initio* structure solution of MIL-53*as*-ht-vrt is obtained with the space group *Imma* with a = 6.65(3) Å, b = 17.27(3) Å, c = 12.54(5) Å. Compared with MIL-53*lt*-v, there are no slight diffuse streaks from projections of MIL-53*as*-ht-vrt, which might also call for special attention for EM experiments of guests-adsorbed materials.

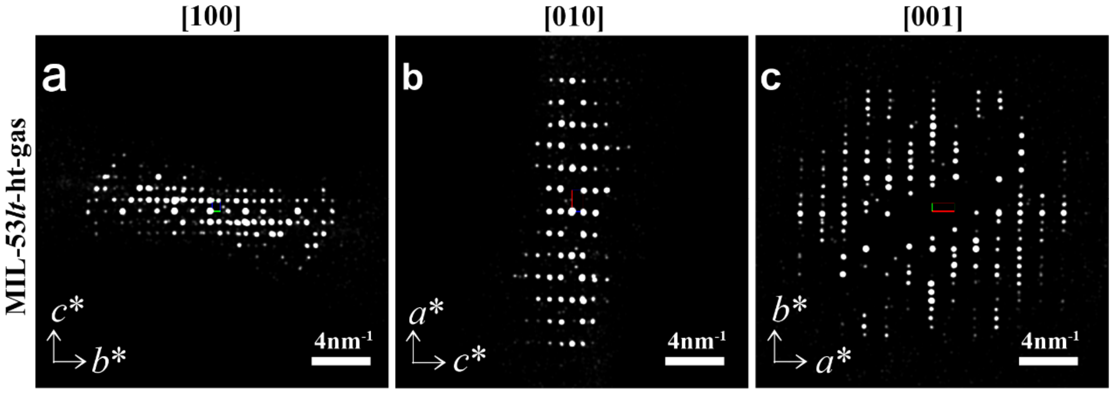

Supplementary Figure 27 | The projections of 3D ED data from MIL-53*lt*-ht-gas under the conditions of 0.1 MPa air and 603 K along (a) [100], (b) [010] and (c) [001] directions respectively.

For MIL-53*lt*-ht-gas, the observed reflection conditions are summarized as *hkl: h* + *k* + *l* = 2*n*; 0*kl: k* + *l* = 2*n*; *h*0*l: h* + *l* = 2*n* and *hk*0: *h* , *k* = 2n, which suggest two possible space groups: *Imma* (no. 74) and *Im*2*a* (no. 46). Using direct methods, *ab initio* structure solution of MIL-53*lt*-ht-gas is obtained with the space group *Imma* and *a* = 6.62(2) Å, *b* = 16.73(4) Å, *c* = 12.89(4) Å.

Section 2 | Supplementary Tables

Supplementary Table 1 | The structure solution and refinement statistic of MIL-53*lt*-cryo.

**MIL-53*lt*-cryo**	
Environment	~100 K, <1×10^{-5} Pa
Molecular	$Al(OH)[O_2C\text{-}C_6H_4\text{-}CO_2]\cdot[H_2O]_1$
Tilt range (°)	90
Datasets merged	4
Radiation source	200 kV Electron
Beam dose ($e^-Å^{-2}s^{-1}$)	~ 0.045
Space group	$P2_1/n$
a (Å)	19.55(8)
b (Å)	15.01(3)
c (Å)	6.655(10)
β (°)	103.7(2)
V ($Å^3$)	1898(9)
Resolution (Å)	0.80
R_{int}	0.2350
R_{meas}	0.270
$I/\sigma(I)$	5.7
$CC_{1/2}$	97.7
Completeness (%)	99.6
Total reflections	22146
Unique reflections	3846
No. parameters	130
No. restraints	0
R_1 ($F_o > 4\sigma(F_o)$)	0.1939
R_1 all	0.2320
wR_2	0.5014
GooF	1.524
CCDC No.	2124556

Supplementary Table 2 | Fractional atomic coordinates for MIL-53*lt*-cryo.

MIL-53*lt*-cryo

Space group: $P2_1/n$ (no. 14); a = 19.55(8)Å; b = 15.01(3)Å; c = 6.655(10)Å; $\alpha = \gamma = 90^{\circ}$, β = 103.7(2)°; V = 1898(9)Å^3; Z = 4

Atom	*x*	*y*	*z*	Uiso (Å^2)
Al1	0.74639(17)	0.6249(2)	0.5115(5)	0.0152(10)
Al2	0.74684(16)	0.6240(2)	1.0104(5)	0.0128(9)
O5	0.7489(2)	0.5689(4)	0.2624(6)	0.0139(12)
O10	0.8338(2)	0.5648(3)	0.6431(7)	0.0141(11)
O3	0.6600(2)	0.6848(3)	0.3787(7)	0.0154(11)
O7	0.6644(2)	0.5547(3)	0.8803(7)	0.0154(11)
O2	0.3296(2)	0.8061(3)	-0.3605(7)	0.0156(11)
O8	0.7444(2)	0.6788(4)	0.7599(6)	0.0131(11)
O9	0.8119(2)	0.5368(3)	0.9556(7)	0.0133(11)
O1	0.3035(3)	0.7789(3)	-0.0515(8)	0.0165(11)
C1	0.3457(3)	0.7813(4)	-0.1709(9)	0.0152(14)
O6	0.6901(2)	0.5282(3)	0.5736(8)	0.0157(11)
O4	0.6815(2)	0.7105(3)	0.0634(8)	0.0156(11)
C8	0.6412(3)	0.7094(4)	0.1910(9)	0.0168(14)
O12	0.7791(3)	0.3865(4)	0.1966(9)	0.0104(18)
H12A	0.753305	0.427686	0.226528	0.016
H12B	0.791068	0.405501	0.089584	0.016
O11	0.7209(3)	0.8621(4)	0.8261(9)	0.0098(19)
H11A	0.74047	0.829166	0.752314	0.015
H11B	0.706844	0.826103	0.906014	0.015
C13	0.8535(3)	0.5408(4)	0.8307(9)	0.0147(14)
C12	0.6475(3)	0.5315(4)	0.6898(9)	0.0159(14)
C2	0.4215(3)	0.7607(4)	-0.0791(10)	0.0179(14)
C6	0.5433(3)	0.7562(4)	-0.1077(9)	0.0171(14)
H6	0.576392	0.762376	-0.186644	0.021
C5	0.5162(3)	0.7217(4)	0.2255(9)	0.0142(13)
H5	0.531054	0.705873	0.364045	0.017
C10	0.5504(3)	0.4882(4)	0.3813(10)	0.0175(14)
H10	0.583957	0.480319	0.304476	0.021
C15	0.9522(3)	0.4920(4)	1.1286(10)	0.0193(15)
H15	0.920091	0.486369	1.211056	0.023
C9	0.5214(3)	0.5236(4)	0.7135(9)	0.0142(14)
H9	0.535664	0.538785	0.852662	0.017
C16	0.9759(3)	0.5242(4)	0.7881(10)	0.0166(14)
H16	0.959729	0.539413	0.649502	0.02
C3	0.4440(3)	0.7368(4)	0.1328(10)	0.0194(14)
H3	0.410966	0.730885	0.211963	0.023
C4	0.4724(3)	0.7717(4)	-0.2005(10)	0.0178(14)
H4	0.458515	0.788891	-0.338497	0.021

C14	0.9288(3)	0.5169(4)	0.9176(9)	0.0155(14)
C11	0.5718(3)	0.5119(4)	0.5920(9)	0.0155(14)
C7	0.5658(3)	0.7319(4)	0.0979(10)	0.0175(14)

Supplementary Table 3 | The structure solution and refinement statistic of MIL-53*lt*-gas (air).

MIL-53*lt*-gas (air)	
Environment	298K, air 0.1MPa
Molecular	$Al(OH)[O_2C\text{-}C_6H_4\text{-}CO_2]\cdot[H_2O]_1$
Tilt range (°)	45
Datasets merged	10
Radiation source	200 kV Electron
Beam dose ($e^- Å^{-2} s^{-1}$)	~ 0.045
Space group	*Cc*
a (Å)	19.55(2)
b (Å)	7.81(2)
c (Å)	6.62(2)
β (°)	104.5(2)
V ($Å^3$)	978(4)
Resolution (Å)	0.80
R_{int}	0.2371
R_{meas}	0.263
$I/\sigma(I)$	5.4
$CC_{1/2}$	98.4
Completeness (%)	71.1
Total reflections	6786
Unique reflections	1388
No. parameters	65
No. restraints	33
R_1 ($F_o > 4\sigma(F_o)$)	0.1383
R_1 all	0.1682
wR_2	0.3602
GooF	1.161
CCDC No.	2124554

Supplementary Table 4 | Fractional atomic coordinates for MIL-53*lt*-gas (air).

MIL-53*lt*-gas (air)

Space group: *Cc* (no. 9); a = 19.55(2)Å; b = 7.81(2)Å; c = 6.62(2)Å; $\alpha = \gamma$ = 90˚, β = 104.5(2)˚; V =978(4) Å^3; Z = 4

Atom	*x*	*y*	*z*	Uiso (Å^2)
Al1	0.1658(4)	0.9895(17)	0.7621(12)	0.0103(13)
C2	0.0590(5)	0.832(3)	0.4272(14)	0.018(3)
O5	0.1001(4)	0.826(2)	0.3132(12)	0.0138(17)
C4	0.9338(6)	0.813(4)	0.453(2)	0.045(5)
H4	0.948187	0.861867	0.585509	0.054
C3	0.9841(5)	0.779(2)	0.3388(15)	0.014(3)
C8	0.9603(5)	0.744(3)	0.1258(14)	0.015(3)
H8	0.991852	0.746093	0.041333	0.019
C7	0.8893(4)	0.705(2)	0.0395(12)	0.008(3)
H7	0.874077	0.671253	-0.099091	0.010
C6	0.8416(6)	0.719(2)	0.1638(16)	0.031(4)
C5	0.8627(6)	0.776(3)	0.3696(16)	0.028(4)
H5	0.830060	0.788283	0.449413	0.033
O4	0.0806(4)	0.871(2)	0.6136(12)	0.0138(17)
O2	0.1648(6)	0.8899(16)	0.0081(18)	0.0153(17)
O1	0.2481(5)	0.126(2)	0.8827(14)	0.019(2)
O3	0.2247(5)	0.820(3)	0.7056(15)	0.024(3)
C1	0.2643(5)	0.178(3)	0.0765(16)	0.013(3)
O6	0.1839(16)	0.522(8)	0.945(5)	0.061(11)
H6A	0.14704	0.463895	0.945087	0.091
H6B	0.193547	0.49636	0.830439	0.091

Supplementary Table 5 | The structure solution and refinement statistic of MIL-53*lt*-liquid (H_2O).

MIL-53*lt*- liquid (H_2O)*	
Environment	298 K, distilled water
Molecular	Al(OH)[O_2C-C_6H_4-CO_2] [H_2O]$_1$
Tilt range (°)	45
Datasets merged	/
Radiation source	200 kV Electron
Beam dose ($e^{-}Å^{-2}s^{-1}$)	~ 0.045
Space group	*Cc*
a (Å)	19.54
b (Å)	7.62
c (Å)	6.56
β (°)	105.0
V (Å^3)	943.47
Resolution (Å)	1.0
R_{int}	0.2455
$I/\sigma(I)$	2.1
Completeness (%)	20.8
Total reflections	330
Unique reflections	143
R_1 ($F_o > 4\sigma(F_o)$)	0.3094
wR_2 (%)	0.7164
CCDC No.	2124553

Note: * means the complete reflection conditions cannot be summarized from experimental 3D ED data and standard uncertainties of unit cell parameters are not provided due to the low completeness and low signal to noise ratio. The space group is assumed to be same as MIL-53*lt*-gas (air).

Supplementary Table 6 | Fractional atomic coordinates for MIL-53*lt*-liquid (H_2O).

MIL-53*lt*-liquid (H_2O)*				
Space group: *Cc* (no. 9); *a* = 19.54Å; *b* = 7.62Å; *c* = 6.56Å; $\alpha = \gamma = 90^\circ$, $\beta = 105^\circ$; *V* = 943.47 Å^3; *Z* = 4				
Atom	*x*	*y*	*z*	Uiso (Å^2)
O6	0.141(3)	0.59(4)	0.532(7)	0.03
Al1	0.171(3)	0.947(11)	0.793(7)	0.03
C2	0.072(3)	0.794(11)	0.439(8)	0.03
O5	0.116(3)	0.825(13)	0.329(7)	0.03
C4	0.947(3)	0.773(12)	0.453(9)	0.03
C3	0.996(4)	0.762(11)	0.333(8)	0.03
C8	0.975(4)	0.718(14)	0.120(9)	0.03
C7	0.905(4)	0.685(15)	0.028(10)	0.03
C6	0.855(4)	0.696(15)	0.148(11)	0.03
C5	0.876(4)	0.740(14)	0.360(10)	0.03
O4	0.085(3)	0.847(10)	0.631(7)	0.03
O2	0.178(4)	0.815(13)	0.055(7)	0.03
O1	0.257(3)	0.059(12)	0.925(8)	0.03
O3	0.238(4)	0.766(12)	0.721(9)	0.03
C1	0.276(3)	0.125(14)	0.110(9)	0.03

Note: * means the complete reflection conditions cannot be summarized from experimental 3D ED data due to the low completeness. The space group is assumed to be same as MIL-53*lt*-gas (air).

Supplementary Table 7 | The structure solution and refinement statistic of MIL-53*as*-v.

MIL-53*as*-v	
Environment	298K, <1×10^{-5}Pa
Molecular	$Al(OH)[O_2C\text{-}C_6H_4\text{-}CO_2]\cdot[HO_2C\text{-}C_6H_4\text{-}CO_2H]_{0.55}$*
Tilt range (°)	70-110
Datasets merged	2
Radiation source	200 kV Electron
Beam dose ($e^-Å^{-2}s^{-1}$)	~ 0.045
Space group	*Pnma*
a (Å)	17.03(1)
b (Å)	6.57(2)
c (Å)	12.16(2)
V ($Å^3$)	1361(5)
Resolution (Å)	0.85
R_{int}	0.1935
R_{meas}	0.2150
$I/\sigma(I)$	7.9
$CC_{1/2}$	98.8
Completeness (%)	99.1
Total reflections	8238
Unique reflections	1261
No. parameters	100
No. restraints	162
R_1 ($F_o > 4\sigma(F_o)$)	0.1900
R_1 all	0.2177
wR_2	0.4768
GooF	1.717
CCDC No.	2124555

*0.55 is the result of TGA of MIL-53*as*.

Supplementary Table 8 | Fractional atomic coordinates for MIL-53*as*-v.

MIL-53*as*-v

Space group: *Pnma* (no. 62); a = 17.03(1)Å; b = 6.57(2)Å; c =12.16(2)Å; $\alpha = \beta = \gamma = 90^{\circ}$; V = 1361(5) Å^3; Z = 8

Atom	*x*	*y*	*z*	Uiso (Å^2)
Al1	0.500000	0.500000	0.000000	0.0165(14)
O1	0.4948(3)	0.750000	0.9334(6)	0.0177(17)
O3	0.4274(3)	0.5797(8)	0.1103(5)	0.0302(16)
C4	0.3999(4)	0.750000	0.1368(8)	0.028(2)
O2	0.4135(3)	0.4210(8)	0.9134(5)	0.0298(16)
C5	0.3277(4)	0.750000	0.2066(7)	0.028(2)
C2	0.3085(4)	0.250000	0.8282(8)	0.028(2)
C3	0.3841(4)	0.250000	0.8904(7)	0.022(2)
C1	0.2752(3)	0.0678(8)	0.7972(6)	0.044(2)
H1	0.298941	0.945247	0.816295	0.052
C6	0.2938(3)	0.9310(8)	0.2374(6)	0.047(2)
H6	0.317382	0.053437	0.217813	0.056
C8	0.4712(14)	0.166(4)	0.647(2)	0.107(13)
C7	0.423(3)	0.250000	0.579(4)	0.089(18)
C9	0.4688(19)	0.831(5)	0.649(3)	0.22(2)
C12	0.3633(17)	0.250000	0.533(3)	0.112(14)
C11	0.4128(19)	0.002(5)	0.585(3)	0.23(2)
C10	0.3732(19)	0.750000	0.544(3)	0.219(14)

Supplementary Table 9 | Anisotropic atomic displacement parameters of MIL-53*as*-v.

MIL-53*as*-v

Space group: *Pnma* (no. 62); a = 17.03(1)Å; b = 6.57(2)Å; c =12.16(2)Å; $\alpha = \beta = \gamma$ = 90˚; V = 1361(5) Å^3; Z = 8

Atom	U_{11} (Å^2)	U_{22} (Å^2)	U_{33} (Å^2)	U_{23} (Å^2)	U_{13} (Å^2)	U_{12} (Å^2)
Al1	0.015(2)	0.015(3)	0.019(2)	0.000(2)	-0.0009(16)	0.0011(15)
O1	0.016(3)	0.020(4)	0.018(4)	0.000	0.002(2)	0.000
O3	0.027(3)	0.022(3)	0.042(3)	0.004(3)	0.020(2)	0.0051(18)
C4	0.018(4)	0.029(4)	0.035(5)	0.000	0.021(3)	0.000
O2	0.022(2)	0.023(3)	0.045(3)	0.004(3)	-0.012(2)	-0.0021(17)
C5	0.020(4)	0.026(5)	0.037(5)	0.000	0.022(3)	0.000
C2	0.020(4)	0.024(4)	0.041(5)	0.000	-0.015(3)	0.000
C3	0.017(3)	0.023(4)	0.027(5)	0.000	-0.008(3)	0.000
C1	0.036(4)	0.016(4)	0.078(6)	0.000(4)	-0.037(4)	0.004(2)
C6	0.033(3)	0.023(4)	0.083(6)	-0.007(4)	0.042(4)	-0.007(3)

Supplementary Table 10 | The structure solution and refinement statistic of MIL-53*as*-ht-v.

**MIL-53*as*-ht-v**	
Environment	603 K, $<1\times10^{-5}$ Pa
Molecular	$Al(OH)[O_2C-C_6H_4-CO_2]$
Tilt range (°)	45
Datasets merged	5
Radiation source	200 kV Electron
Beam dose ($e^{-}Å^{-2}s^{-1}$)	~ 0.01
Space group	*Imma*
a (Å)	6.61(3)
b (Å)	17.25(3)
c (Å)	12.81(1)
V ($Å^3$)	1461(7)
Resolution (Å)	0.81
R_{int}	0.2451
R_{meas}	0.277
$I/\sigma(I)$	5.2
$CC_{1/2}$	93.5
Completeness (%)	99.7
Total reflections	5218
Unique reflections	818
No. parameters	40
No. restraints	16
R_1 ($F_o > 4\sigma(F_o)$)	0.1932
R_1 all	0.2195
wR_2	0.4845
GooF	1.567
CCDC No.	2168111

Supplementary Table 11 | Fractional atomic coordinates for MIL-53*as*-ht-v.

MIL-53*as*-ht-v

Space group: *Imma* (no. 74); a = 6.61(3)Å; b = 17.25(3)Å; c = 12.81(1)Å; $\alpha = \beta = \gamma = 90^{\circ}$; V = 1461(7) Å^3; Z = 16

Atom	*x*	*y*	*z*	Uiso (Å^2)
Al1	0.250000	0.750000	0.250000	0.0527(17)
O1	0.500000	0.750000	0.1853(6)	0.069(3)
C1	0.500000	0.6404(4)	0.3725(6)	0.074(3)
C2	0.500000	0.5681(5)	0.4361(7)	0.086(3)
O2	0.3309(10)	0.6694(4)	0.3469(4)	0.087(2)
C3	0.6803(13)	0.5327(5)	0.4695(7)	0.136(4)
H3	0.803354	0.554233	0.449522	0.163

Supplementary Table 12 | Anisotropic atomic displacement parameters of MIL-53*as*-ht-v.

MIL-53*as*-ht-v

Space group: *Imma* (no. 74); a = 6.61(3)Å; b = 17.25(3)Å; c = 12.81(1)Å; $\alpha = \beta = \gamma$ = 90˚; V = 1461(7)Å^3; Z = 16

Atom	U_{11} (Å^2)	U_{22} (Å^2)	U_{33} (Å^2)	U_{23} (Å^2)	U_{13} (Å^2)	U_{12} (Å^2)
Al1	0.062(4)	0.025(2)	0.071(3)	0.000	-0.002(3)	0.000
O1	0.073(6)	0.066(5)	0.068(4)	0.000	0.000	0.000
C1	0.076(7)	0.053(4)	0.095(5)	0.034(4)	0.000	0.000
C2	0.072(6)	0.061(5)	0.126(6)	0.047(4)	0.000	0.000
O2	0.074(4)	0.075(3)	0.110(4)	0.043(3)	-0.003(3)	0.001(3)
C3	0.074(5)	0.120(6)	0.212(8)	0.124(6)	0.011(5)	-0.001(5)

Supplementary Table 13 | The structure solution and refinement statistic of MIL-53*as*-ht-vrt.

MIL-53*as*-ht-vrt	
Environment	298 K, <1×10^{-5} Pa
Molecular	Al(OH)[O_2C-C_6H_4-CO_2]
Tilt range (°)	45
Datasets merged	5
Radiation source	200 kV Electron
Beam dose (e^{-}Å^{-2}s^{-1})	~ 0.045
Space group	*Imma*
a (Å)	6.65(3)
b (Å)	17.27(3)
c (Å)	12.54(5)
V (Å^3)	1440(9)
Resolution (Å)	0.80
R_{int}	0.1800
R_{meas}	0.2120
I/σ(I)	7.0
$CC_{1/2}$	97.5
Completeness (%)	96.5
Total reflections	4943
Unique reflections	795
No. parameters	40
No. restraints	0
R_1 ($F_o > 4\sigma(F_o)$)	0.1876
R_1 all	0.1979
wR_2	0.4465
GooF	1.162
CCDC No.	2167321

Supplementary Table 14 | Fractional atomic coordinates for MIL-53*as*-ht-vrt.

MIL-53*as*-ht-vrt

Space group: *Imma* (no. 74); a = 6.65(3)Å; b = 17.27(3)Å; c = 12.54(5)Å; $\alpha = \beta = \gamma = 90^{\circ}$; V = 1440(9)Å^3; Z = 16

Atom	*x*	*y*	*z*	Uiso (Å^2)
Al1	0.250000	0.250000	0.750000	0.0324(16)
O1	0.000000	0.250000	0.8145(8)	0.037(2)
C1	0.000000	0.1415(5)	0.6279(7)	0.042(2)
C2	0.000000	0.0680(5)	0.5638(8)	0.053(2)
O2	0.1679(8)	0.1692(3)	0.6530(4)	0.0537(17)
C3	-0.1799(10)	0.0338(4)	0.5301(7)	0.081(3)
H3	-0.301701	0.056624	0.548448	0.098

Supplementary Table 15 | Anisotropic atomic displacement parameters of MIL-53*as*-ht-vrt.

MIL-53*as*-ht-vrt

Space group: *Imma* (no. 74); a = 6.65(3)Å; b = 17.27(3)Å; c = 12.54(5)Å; $\alpha = \beta = \gamma = 90^{\circ}$; V = 1440(9)Å^3; Z = 16

Atom	U_{11} (Å^2)	U_{22} (Å^2)	U_{33} (Å^2)	U_{23} (Å^2)	U_{13} (Å^2)	U_{12} (Å^2)
Al1	0.038(3)	0.024(3)	0.036(3)	0.000	-0.003(3)	0.000
O1	0.034(4)	0.031(4)	0.047(5)	0.000	0.000	0.000
C1	0.035(4)	0.037(4)	0.054(5)	-0.009(4)	0.000	0.000
C2	0.036(4)	0.049(5)	0.073(6)	-0.018(5)	0.000	0.000
O2	0.045(3)	0.046(3)	0.070(4)	-0.025(3)	0.000(2)	-0.009(2)
C3	0.037(3)	0.073(5)	0.134(8)	-0.061(5)	0.006(4)	0.005(3)

Supplementary Table 16 | The structure solution and refinement statistic of MIL-53*lt*-ht-gas.

MIL-53*lt*-ht-gas	
Environment	603 K, 0.1MPa air
Molecular	$Al(OH)[O_2C\text{-}C_6H_4\text{-}CO_2]$
Tilt range (°)	45
Datasets merged	2
Radiation source	200 kV Electron
Beam dose ($e^{-}Å^{-2}s^{-1}$)	~ 0.01
Space group	*Imma*
a (Å)	6.62(2)
b (Å)	16.73(4)
c (Å)	12.89(4)
V ($Å^3$)	1428(7)
Resolution (Å)	0.80
R_{int}	0.2438
R_{meas}	0.296
$I/\sigma(I)$	4.4
$CC_{1/2}$	98.4
Completeness (%)	74.1
Total reflections	2134
Unique reflections	605
No. parameters	40
No. restraints	94
R_1 ($F_o > 4\sigma(F_o)$)	0.1964
R_1 all	0.2903
wR_2	0.4739
GooF	1.303
CCDC No.	2167322

Supplementary Table 17 | Fractional atomic coordinates for MIL-53*lt*-ht-gas.

MIL-53*lt*-ht-gas

Space group: *Imma* (no. 74); a = 6.62(2)Å; b = 16.73(4)Å; c = 12.89(4)Å; $\alpha = \beta = \gamma = 90^{\circ}$; V = 1428(7)Å^3; Z = 16

Atom	*x*	*y*	*z*	Uiso (Å^2)
Al1	0.750000	0.750000	0.750000	0.033(3)
O1	0.000000	0.750000	0.8104(16)	0.037(4)
C1	0.000000	0.8591(6)	0.6310(14)	0.058(5)
C2	0.000000	0.9316(7)	0.5605(16)	0.065(5)
O2	0.8340(9)	0.8313(4)	0.6538(9)	0.055(4)
C3	0.1771(11)	0.9655(5)	0.5297(12)	0.101(6)
H3	0.299086	0.942141	0.548778	0.121

Supplementary Table 18 | Anisotropic atomic displacement parameters of MIL-53*lt*-ht-gas.

MIL-53*lt*-ht-gas						
Space group: *Imma* (no. 74); a = 6.62(2)Å; b = 16.73(4)Å; c = 12.89(4)Å; $\alpha = \beta = \gamma$ = 90˚; V = 1428(7)Å^3; Z = 16						
Atom	U_{11} (Å^2)	U_{22} (Å^2)	U_{33} (Å^2)	U_{23} (Å^2)	U_{13} (Å^2)	U_{12} (Å^2)
Al1	0.025(3)	0.025(4)	0.050(10)	0.000	0.002(5)	0.000
O1	0.023(4)	0.049(7)	0.038(13)	0.000	0.000	0.000
C1	0.031(4)	0.048(6)	0.095(16)	0.036(7)	0.000	0.000
C2	0.032(4)	0.070(7)	0.094(16)	0.053(8)	0.000	0.000
O2	0.035(3)	0.068(5)	0.062(10)	0.036(5)	0.003(4)	-0.004(3)
C3	0.036(3)	0.116(8)	0.151(18)	0.103(9)	0.000(7)	0.004(4)

Section 3 | Supplementary References

1. Spek, A. L. Single-crystal structure validation with the program PLATON. *J. Appl. Cryst.* **36**, 7-13 (2003).
