# Supplementary material for: Atomic-level structural responsiveness to environmental conditions from 3D electron diffraction
Source: Nat Commun. 2022 Nov 4;13:6625. doi: 10.1038/s41467-022-34237-1 (PMC9636419; doi:10.1038/s41467-022-34237-1)
Supplement: Supplementary file 2 — Description of Additional Supplementary Files [file 41467_2022_34237_MOESM2_ESM.docx]

**Description of Additional Supplementary Files**

File Name: Supplementary Movie 1

Description: Droplets aggregate during beam irradiation.

File Name: Supplementary Movie 2

Description: Electron diffraction patterns show the random rotation of a MIL-53*lt* particle suspended in water layer.
